# Shared decision making of burdensome surveillance tests using personalized schedules and their burden and benefit

**DOI:** 10.1002/sim.9347

**Published:** 2022-02-10

**Authors:** Anirudh Tomer, Daan Nieboer, Monique J. Roobol, Ewout W. Steyerberg, Dimitris Rizopoulos

**Affiliations:** ^1^ Department of Biostatistics Erasmus University Medical Center Rotterdam Zuid‐Holland The Netherlands; ^2^ Department of Public Health Erasmus University Medical Center Rotterdam Zuid‐Holland The Netherlands; ^3^ Department of Urology Erasmus University Medical Center Rotterdam Zuid‐Holland The Netherlands; ^4^ Department of Biomedical Data Sciences Leiden University Medical Center Leiden Zuid‐Holland The Netherlands

**Keywords:** chronic NCDs, invasive diagnostic tests, joint models, personalized schedules, shared decision making

## Abstract

Benchmark surveillance *tests* for detecting disease *progression* (eg, biopsies, endoscopies) in early‐stage chronic noncommunicable diseases (eg, cancer, lung diseases) are usually burdensome. For detecting progression timely, patients undergo invasive tests planned in a fixed one‐size‐fits‐all manner (eg, annually). We aim to present personalized test schedules based on the risk of disease progression, that optimize the burden (the number of tests) and the benefit (shorter time delay in detecting progression is better) better than fixed schedules, and enable shared decision making. Our motivation comes from the problem of scheduling biopsies in prostate cancer surveillance. Using joint models for time‐to‐event and longitudinal data, we consolidate patients' longitudinal data (eg, biomarkers) and results of previous tests, into individualized future cumulative‐risk of progression. We then create personalized schedules by planning tests on future visits where the predicted cumulative‐risk is above a *threshold* (eg, 5% risk). We update personalized schedules with data gathered over follow‐up. To find the optimal risk threshold, we minimize a utility function of the expected number of tests (burden) and expected time delay in detecting progression (shorter is beneficial) for different thresholds. We estimate these two in a patient‐specific manner for following any schedule, by utilizing a patient's predicted risk profile. Patients/doctors can employ these quantities to compare personalized and fixed schedules objectively and make a shared decision of a test schedule.

## 1 INTRODUCTION

**FIGURE 1:**
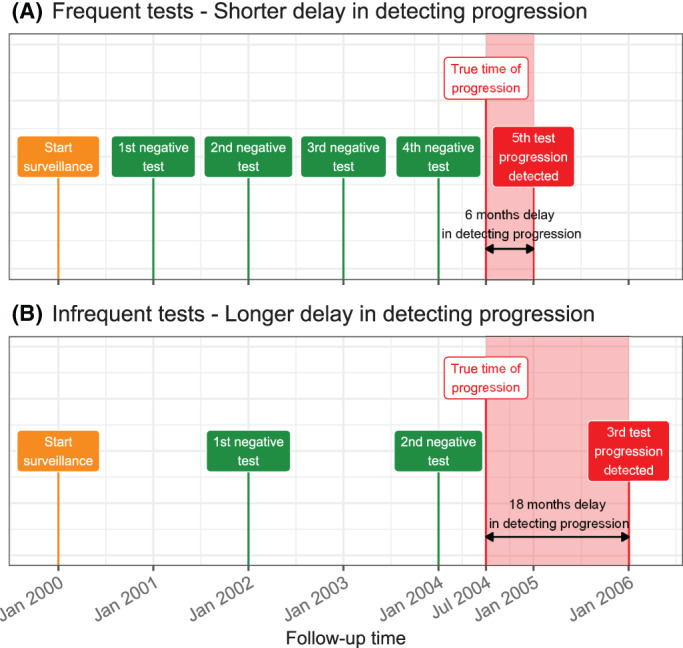
Goal: Finding the optimal tradeoff between the number of invasive tests (burden) and time delay in detecting progression (shorter is beneficial). A progression is a nonterminal event in the surveillance of early‐stage chronic noncommunicable diseases. The true time of progression for the patient illustrated in this figure is July 2004. Since invasive tests are conducted repeatedly, progression is interval‐censored and always observed with a delay. Frequent periodical invasive tests in (A) lead to a shorter time delay in detecting progression than infrequent periodical invasive tests in (B). The interval‐censored time of progression is Jan 2004 to Jan 2005 in (A) and between Jan 2004 and Jan 2006 in (B)

Chronic noncommunicable diseases (eg, cancer, lung, cardiovascular diseases) cause 60% to 70% of human deaths worldwide.^1^ Often patients diagnosed with an early‐stage disease undergo surveillance *tests* to detect disease *progression* timely. A progression is a nonterminal event, and usually a trigger for treatment and/or removal from surveillance. Benchmark tests used for confirming progression are usually *invasive*, for example, biopsies in prostate cancer surveillance,^2^ endoscopies in Barrett's esophagus,^3^ colonoscopies in colorectal cancer,^4^ and bronchoscopies in post lung transplant^5^ surveillance. These invasive tests are repeated until progression is observed, typically as per a one‐size‐fits‐all *fixed schedule*, for example, biannually.^2^, ^4^, ^5^ A time gap between tests causes a time delay in detecting progression (Figure 1). A shorter delay in detecting progression (*benefit*) can provide a larger window of opportunity for curative treatment. However, with fixed schedules, this means conducting tests frequently. Frequent tests are *burdensome* as they may cause pain and/or severe medical complications.^4^, ^6^ Consequently, patients may not always comply with frequent tests.^2^, ^7^ In general, because fixed schedules do not differentiate between fast and slow/nonprogressing patients, they impose disproportionate burden/benefits across the patient population.

The goal of this work is two‐fold. First, to create *personalized* invasive test schedules that better optimize the burden (number of invasive tests) and benefit (shorter delay in detecting progression) of invasive tests than fixed schedules (Figure 1). To this end, we intend to use the patients' clinical data accumulated over the surveillance follow‐up. This data includes baseline characteristics, previous test results, and longitudinal outcomes (eg, biomarkers, medical imaging, physical examination). Second, we develop a methodology to estimate the burden (number of invasive tests) and benefit (shorter time delay in detecting progression) of invasive test schedules. We intend to use these criteria for comparing test schedules objectively and subsequently enable shared decision making while choosing a test schedule.

The idea of personalizing the invasive test schedules is not new. In fact, currently, some surveillance protocols personalize test schedules using heuristic methods such as decision flowcharts.^2^, ^3^ However, flowcharts discretize continuous outcomes, often exploit only the last measurement, ignore the measurement error in observed data, and plan only one test at a time. Alternatively, a complete personalized schedule of tests can be obtained using partially observable Markov decision processes or POMDPs.^8^, ^9^ Although, POMDPs typically discretize continuous longitudinal outcomes to avoid the curse of dimensionality. Besides, in scenarios such as ours, where decisions (test/no test) and disease state (low‐grade disease/progressed) are both binary, POMDPs may not be necessary either. The reason is that such POMDPs give the same optimal schedule, which can be alternatively obtained by just planning a test when the probability of transition from nonprogressed to progressed state is more than a certain threshold^10^ ^(equation 1)^.

Personalized schedules have also been obtained by optimizing an explicit utility function of the burden and/or benefit of a schedule. A challenge in this approach is quantifying burden and benefit. In our previous work,^11^ we quantified the burden and benefit as the time difference by which a future test undershoots (unnecessary test) or overshoots (delayed detection) the true progression time of a patient, respectively. These choices limited us to plan only one future test at a time. Others^12^ proposed obtaining a complete test schedule by quantifying the burden of a test schedule as the expected number of tests and benefit as expected time delay in detecting progression. To obviate the issue that the number of tests and delay have different scales and units, they proposed scheduling tests when the risk of progression is above a threshold. Schedules based on risk threshold have also been proposed previously.^10^, ^13^ The clinical interpretation of risk and the choice of risk threshold is not straightforward. In our previous work on risk‐based test schedules^14^ we, and others,^15^ motivated the choice of risk threshold to be based on measures of diagnostic accuracy (eg, false positive rate, true positive rate). However, measures of diagnostic accuracy are not personalized criteria for choosing risk thresholds. Besides, a single risk‐based test decision does not inform patients about future clinical consequences of continuing on surveillance.

In this article, we make two significant improvements over our own two previous works on the same topic.^11^, ^14^ First, instead of planning one test at a time, we derive full risk‐based personalized test schedules. Thus, at any follow‐up visit, patients know the time of all future tests planned for them. The personalized schedules also dynamically update with new clinical data over follow‐up. Second, along with each schedule, we provide patients the clinical consequences of following it. Namely, the expected number of tests required out of all planned tests to detect progression and the expected time delay in detecting progression. There are three advantages of using these two criteria for schedule selection instead of measures of diagnostic accuracy previously proposed by us^14^ and others.^15^ First, by using our proposed criteria we can evaluate the performance of a complete schedule and not just a single test decision. Second, the proposed criteria are easily‐quantifiable surrogates for important clinical aspects such as the window of opportunity for curative treatment, risk of adverse outcomes due to delayed detection of progression, financial costs of tests, risk of side‐effects, and reduction in quality of life, etc. Third, we calculate the expected number of tests and delay in a personalized manner, an improvement over previous work by others.^12^ Hence, for any schedule, fixed or personalized, patients can objectively compare the clinical consequences of opting for them. This can enable shared decision making of invasive test schedules.

The basic idea behind our new approach is as follows. We first develop a full specification of the joint distribution of the patient‐specific longitudinal outcomes and the time of progression. To this end, we utilize joint models for time‐to‐event and longitudinal data^16^, ^17^ because they are inherently personalized. Specifically, joint models utilize patient‐specific random effects^18^ to model longitudinal outcomes without discretizing them. Subsequently, we input clinical data of a new patient into the fitted model to obtain their predicted patient‐specific cumulative‐risk of progression at future visits. We then create personalized schedules by planning tests on future visits where this predicted cumulative‐risk is above a particular *threshold* (eg, 5% risk). We automate the choice of this threshold and the resulting schedule by optimizing a utility function of the expected number of tests and time delay in detecting progression for personalized schedules. To estimate these two quantities in a patient‐specific manner we use patient's predicted risk profiles. Hence, patients/doctors can compare the consequences of opting for personalized vs fixed schedules objectively.

Our motivation comes from the task of scheduling biopsies in the world's largest prostate cancer surveillance study, called Prostate Cancer Research International Active Surveillance,^2^ or PRIAS. It has 7813 low/very‐low grade cancer patients (1134 progressions, 104 904 longitudinal measurements), many of whom are potentially over‐diagnosed due to prostate‐specific antigen (PSA) based screening.^19^ To reduce subsequent over‐treatment, in surveillance, serious treatments (eg, surgery, radiotherapy) are delayed until progression is observed. Surveillance involves regular monitoring of a patient's PSA (ng/mL), digital rectal examination or DRE (tumor shape/size), and biopsy Gleason grade group.^20^ Among these, a biopsy Gleason grade group ≥ 2 is the reference test for confirming progression. Most often, biopsies are scheduled annually.^21^ However, such a frequent schedule can put an unnecessary burden on patients with slow/nonprogressing cancers and cause noncompliance.^2^ Since prostate cancer has the second‐highest incidence among all cancers in males,^22^ individualized biopsy schedules can reduce the burden of biopsies in numerous patients worldwide.

The remaining paper is as follows. Section 2 introduces the joint modeling framework. The personalized scheduling methodology is described in Section 3, and demonstrated for prostate cancer surveillance patients in Section 4. In Section 5, we compare personalized and fixed schedules via a realistic simulation study based on a joint model fitted to the PRIAS dataset.

## 2 JOINT MODEL FOR TIME‐TO‐PROGRESSION AND LONGITUDINAL OUTCOMES

Let $T_{i}^{*}$ denote the true time of disease progression for the ith patient. Progression is always interval censored $l_{i}<T_{i}^{*}\leq r_{i}$ (Figure 1). Here, $r_{i}$ and $l_{i}$ denote the time of the last and second last invasive tests, respectively, when patients progress. In nonprogressing patients, $l_{i}$ denotes the time of the last test and $r_{i}=\infty$. Assuming *K* types of longitudinal outcomes, let $y_{ki}$ denote the $n_{ki}\times 1$ longitudinal response vector of the k‐th outcome, k∈{1,…,K}. The observed data of all *n* patients is given by ${𝒜}_{n}=\{l_{i},r_{i},y_{1i},\ldots y_{Ki};i=1,\ldots ,n\}$.

### 2.1 Longitudinal subprocess

To model multiple longitudinal outcomes in a unified framework, a joint model employs individual generalized linear mixed submodels.^18^ Specifically, the conditional distribution of the *k*‐th outcome $y_{ki}$ given a vector of patient‐specific random effects $b_{ki}$ is assumed to belong to the exponential family, with linear predictor given by,

$$g_{k}[E\{y_{ki}(t)|b_{ki}\}]=m_{ki}(t)=x_{ki}^{⊤}(t)\beta_{k}+z_{ki}^{⊤}(t)b_{ki},$$

where $g_{k}(\cdot )$ denotes a known one‐to‐one monotonic link function, $y_{ki}(t)$ is the value of the k‐th longitudinal outcome for the i‐th patient at time *t*, and $x_{ki}(t)$ and $z_{ki}(t)$ are the time‐dependent design vectors for the fixed $\beta_{k}$ and random effects $b_{ki}$, respectively. To model the correlation between different longitudinal outcomes, we link their corresponding random effects. Specifically, we assume that the vector of random effects $b_{i}={\left(b_{1i}^{⊤},\ldots ,b_{Ki}^{⊤}\right)}^{⊤}$ follows a multivariate normal distribution with mean zero and variance‐covariance matrix *W*.

### 2.2 Survival subprocess

In the survival subprocess, the hazard of progression $h_{i}(t)$ at a time *t* is assumed to depend on a function of patient and outcome‐specific linear predictors $m_{ki}(t)$ and/or the random effects,

$$h_{i}\{t|{ℳ}_{i}(t),w_{i}(t)\}=h_{0}(t)\exp[\gamma^{⊤}w_{i}(t)+\overset{K}{\underset{k=1}{\sum }}f_{k}\{{ℳ}_{ki}(t),w_{i}(t),b_{ki},\alpha_{k}\}],\;t>0,$$

where $h_{0}(\cdot )$ denotes the baseline hazard, ${ℳ}_{ki}(t)=\{m_{ki}(s)|0\leq s<t\}$ is the history of the k‐th longitudinal process up to *t*, and $w_{i}(t)$ is a vector of exogenous, possibly time‐varying covariates with regression coefficients γ. Functions $f_{k}(\cdot )$, parameterized by vector of coefficients $\alpha_{k}$, specify the features of each longitudinal outcome that are included in the linear predictor of the relative‐risk model.^17^, ^23^, ^24^ Some examples, motivated by the literature (subscripts *k* dropped for brevity), are,

$$\left\{\begin{array}{l} f\{{ℳ}_{i}(t),w_{i}(t),b_{i},\alpha \}=\alpha m_{i}(t),\\ f\{{ℳ}_{i}(t),w_{i}(t),b_{i},\alpha \}=\alpha_{1}m_{i}(t)+\alpha_{2}m_{i}^{\prime }(t),\;\text{with}\;m_{i}^{\prime }(t)=\frac{dm_{i}(t)}{dt}. \end{array}\right.$$

These formulations of f(·) postulate that the hazard of progression at time *t* may depend on the underlying level $m_{i}(t)$ (eg, PSA value in prostate cancer) or on both the level and velocity $m_{i}^{\prime }(t)$ (eg, PSA velocity) of the longitudinal outcome at *t*. Lastly, the baseline hazard $h_{0}(t)$ is modeled flexibly using P‐splines.^25^ The detailed specification of the baseline hazard, and the joint parameter estimation of the longitudinal and relative‐risk submodels using the Bayesian approach are presented in Supplementary Material Section A.

## 3 PERSONALIZED SCHEDULE OF INVASIVE TESTS FOR DETECTING PROGRESSION

### 3.1 Cumulative‐risk of progression

Using the joint model fitted to the training data ${𝒜}_{n}$, we aim to derive a personalized schedule of invasive tests for a new patient *j* with true progression time $T_{j}^{*}$. To this end, our calculations exploit the *cumulative‐risk* function. Let $t<T_{j}^{*}$ be the time of the last conducted test at which progression was not observed. Let $\left\{{𝒴}_{1j}(v),\ldots ,{𝒴}_{Kj}(v)\right\}$ denote the history of observed longitudinal data up to the current visit time *v*. The current visit can be after the last negative test, that is, v≥t (eg, PSA after negative biopsy in prostate cancer). The cumulative‐risk of progression for patient *j* at future time u≥t is then given by,

(1) $$\begin{array}{ll} R_{j}(u|t,v) & =\mathrm{Pr}\{T_{j}^{*}\leq u|T_{j}^{*}>t,{𝒴}_{1j}(v),\ldots ,{𝒴}_{Kj}(v),{𝒜}_{n}\}\\ & =\iint \mathrm{Pr}(T_{j}^{*}\leq u|T_{j}^{*}>t,b_{j},\theta )p\{b_{j}|T_{j}^{*}>t,{𝒴}_{1j}(v),\ldots ,{𝒴}_{Kj}(v),\theta \}p(\theta |{𝒜}_{n})db_{j}d\theta \\ & =\iint \{1-\frac{\mathrm{Pr}(T_{j}^{*}>u|b_{j},\theta )}{\mathrm{Pr}(T_{j}^{*}>t|b_{j},\theta )}\}p\{b_{j}|T_{j}^{*}>t,{𝒴}_{1j}(v),\ldots ,{𝒴}_{Kj}(v),\theta \}p(\theta |{𝒜}_{n})db_{j}d\theta ,\\ & =\iint \{1-\frac{\exp[-\int_{0}^{u}h_{i}\{s|{ℳ}_{i}(s),w_{i}(s)\}ds]}{\exp[-\int_{0}^{t}h_{i}\{s|{ℳ}_{i}(s),w_{i}(s)\}ds]}\}p\{b_{j}|T_{j}^{*}>t,{𝒴}_{1j}(v),\ldots ,{𝒴}_{Kj}(v),\theta \}p(\theta |{𝒜}_{n})db_{j}d\theta . \end{array}$$

**FIGURE 2:**
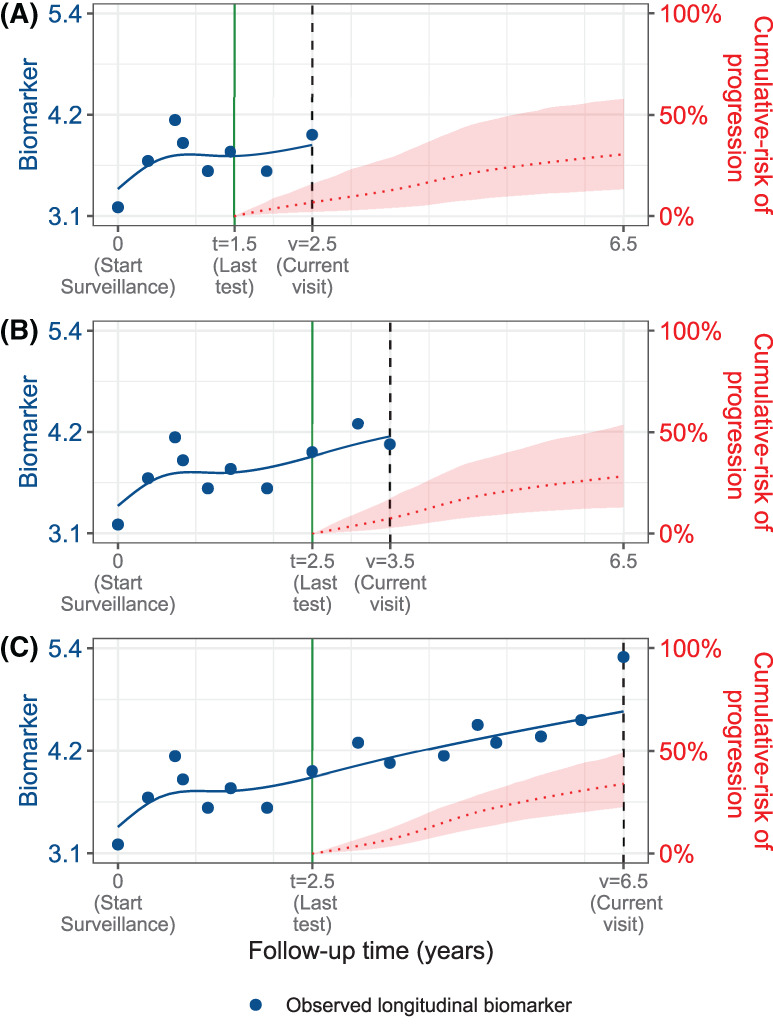
The cumulative‐risk function (1) is time‐dynamic because it automatically updates over time as more longitudinal and invasive test result data becomes available. We illustrate this using a single longitudinal outcome, namely, a continuous biomarker of disease progression (All values are illustrative). (A‐C) are ordered by the time of the current visit *v* (dashed vertical black line) of a new patient. At each of these visits, we combine the accumulated longitudinal data (shown in blue circles), and time of the last negative invasive test *t* (solid vertical green line) to obtain the updated cumulative‐risk profile $R_{j}(u|t,v)$ (dotted red line with 95% credible interval shaded) of the patient. The benefit of this time‐dynamic property is that the resulting schedules in Section 3.2 and their estimated burden and benefit in Section 3.3 are also time‐dynamic

The cumulative‐risk function $R_{j}(\cdot )$ depends on patient‐specific clinical data and the training dataset, via the posterior distribution of the random effects $b_{j}$ and posterior distribution of the vector of all parameters θ of the fitted joint model, respectively. A key property of this cumulative‐risk function is that it is time‐dynamic (illustrated in Figure 2). That is, it automatically updates over time as more longitudinal and invasive test result data becomes available. We next exploit this property to first develop schedules that are also personalized and time‐dynamic, and subsequently to estimate the burden (number of tests required) and benefit (time delay in detecting progression) of the resulting schedules in a time‐dynamic manner.

### 3.2 Personalized test decision rule

In our previous works,^11^, ^14^ we used the cumulative‐risk function in (1) to optimize loss functions inspired from Bayesian decision theory for deciding the time of an invasive test for the *j*th patient. However, this approach assumed that only one test can be conducted for the patient. Consequently, patients could foresee neither the future tests that may be required nor the clinical consequences of opting for such future tests. Hence, in this work we intend to exploit the whole cumulative‐risk profile over time $R_{j}(\cdot )$ to develop full risk‐based personalized schedule of invasive tests. In addition, we aim to use this patient‐specific cumulative‐risk function to estimate the burden (number of tests required) and benefit (time delay in detecting progression) of each schedule we develop for the patients.

Typically, the decision to undergo an invasive test is made on the same visit times on which longitudinal data (eg, biomarkers) are measured. Let $U=\{u_{1},\ldots ,u_{L}\}$ represent a schedule of such visits (eg, biannual PSA measurement in prostate cancer). Here, $u_{1}=v$ is also the current visit time. The maximum future visit time $u_{L}$ can be chosen based on the available information in the training dataset ${𝒜}_{n}$. That is, tests for the new patient *j* are planned only up to a future visit time $u_{L}$ at which a sufficient number of events in ${𝒜}_{n}$ are available for making reliable risk predictions (eg, up to the 80% or 90% percentile of progression times).

**FIGURE 3:**
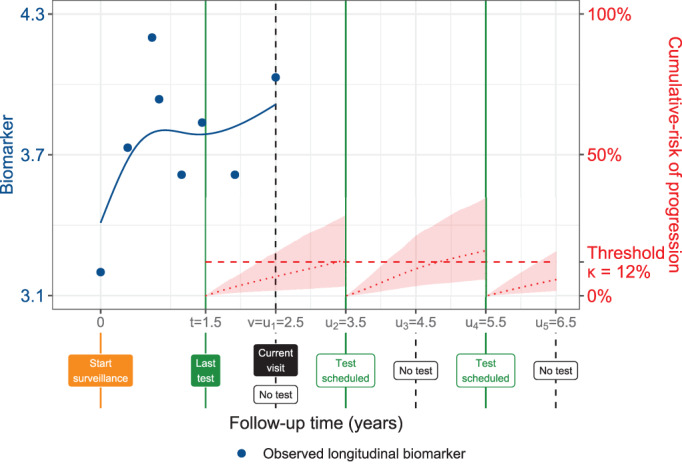
Successive personalized test decisions based on patient‐specific cumulative‐risk of progression (2). Time of current visit: v=2.5 years (dashed vertical black line). Time of the last test on which progression was not observed: t=1.5 years. Longitudinal data up to current visit: ${𝒴}_{j}(v)$ is a continuous biomarker (blue circles). Example risk threshold: κ=0.12 (12%). Grid of future visits on which future tests are planned: U={2.5,3.5,4.5,5.5,6.5} years. The cumulative‐risk profiles $R_{j}(u_{l}|t_{l},v)$ employed in (2) are shown with dotted red lines (95% credible intervals shaded), and are updated each time a test is planned (solid vertical green lines). Future test decisions $Q_{j}(u_{l}|t_{l},v)$ defined in (2) are: $Q_{j}^{\kappa }(u_{1}=2.5|t_{1}=1.5,v)=0$, $Q_{j}^{\kappa }(u_{2}=3.5|t_{2}=1.5,v)=1$, $Q_{j}^{\kappa }(u_{3}=4.5|t_{3}=3.5,v)=0$, $Q_{j}^{\kappa }(u_{4}=5.5|t_{4}=3.5,v)=1$, and $Q_{j}^{\kappa }(u_{5}=6.5|t_{5}=5.5,v)=0$. All values are illustrative

We propose to take the decision of conducting a test at a future visit time $u_{l}\in U$ if the cumulative‐risk of progression at time $u_{l}$ exceeds a certain risk threshold κ (Figure 3). In particular, the test decision at time $u_{l}$ is given by,

(2) $$Q_{j}^{\kappa }(u_{l}|t_{l},v)=I\{R_{j}(u_{l}|t_{l},v)\geq \kappa \},\;0\leq \kappa \leq 1,$$

where I(·) is the indicator function, $R_{j}(u_{l}|t_{l},v)$ is the cumulative‐risk of progression at the current decision time $u_{l}$, and $t_{l}<u_{l}$ is the time of the last test conducted before $u_{l}$. Thus, the future time at which a test will be planned, depends on both the threshold κ and the cumulative‐risk of the patient. Moreover, when a test gets planned at time $u_{l}$, that is, $Q_{j}^{\kappa }(u_{l}|t_{l},v)=1$, then the cumulative‐risk profile is updated before making the next test decision at time $u_{l+1}$ (Figure 3). Specifically, the cumulative‐risk at time $u_{l+1}$ is updated by setting the corresponding time of the last test $t_{l+1}=u_{l}$. This accounts for the possibility that progression may occur after time $u_{l}<T_{j}^{*}$. Hence, the time of last test $t_{l}$ is defined as,

$$t_{l}=\left\{\begin{array}{ll} t, & \text{if}\;l=1,\\ u_{l-1}, & \text{if}\;l\geq 2\;\text{and}\;Q_{j}^{\kappa }(u_{l-1}|t_{l-1},v)=1,\\ t_{l-1}, & \text{if}\;l\geq 2\;\text{and}\;Q_{j}^{\kappa }(u_{l-1}|t_{l-1},v)=0. \end{array}\right.$$

We further illustrate the test scheduling process using Figure 3. In the figure, at the current visit (a real physical visit of a patient) denoted by l=1 the corresponding time of last test $t_{1}$ is set to $t_{1}=t=1.5$. Here, *t* is the time of the last known test, likely extracted from the medical records of the patient. At the current visit l=1 the cumulative‐risk is lower than the set threshold of 12%. Thus, a decision of not conducting a test is taken at current time $u_{1}$, denoted by $Q_{j}^{\kappa }(u_{1}|t_{1},v)=0$. It is important to note at this point all visits with l>1 are future visits that have not yet occurred. At the next visit l=2 (the first future visit), the corresponding time of last test $t_{2}$ is still set to $t_{2}=t=1.5$ because *t* is still the time of the last test. However, at this visit l=2 the cumulative‐risk is more than the set threshold and it is decided to plan a test at this visit, denoted by $Q_{j}^{\kappa }(u_{2}|t_{2},v)=1$. Consequently, at the third visit l=3 (the second future visit), the time of the last test $t_{3}$ switches from *t* to $t_{3}=t_{2}$. The time $t_{2}$ remains as the time of last test until at any future visit a new test is planned again. The process is continued until the last planned visit l=L. We should note that in all future test decisions (visits with l>1), we use only the observed longitudinal data up to the current (real visit) visit time $u_{1}=v$, that is, $\left\{{𝒴}_{1j}(v),\ldots ,Y_{Kj}(v)\right\}$.

### 3.3 Expected number of tests and expected time delay in detecting progression

To facilitate shared‐decision making of invasive tests, we translate our proposed decision rule, that is, the choice of a specific risk threshold κ, into two clinically relevant quantities. First, the number of tests (burden) we expect to perform for patient *j*, and second, if the patient progresses, the time delay (shorter is beneficial) expected in detecting progression. To calculate these two quantities, we first suppose that patient *j* does not progress between his last negative test at time *t* and the maximum future visit time $u_{L}$. Under this assumption, the subset of future visit times in *U* on which a test is planned using (2) results into a personalized schedule of future tests (Figure 3), given by,

(3) $$\{s_{1},\ldots ,s_{N_{j}}\}=\{u_{l}\in U:Q_{j}^{\kappa }(u_{l}|t_{l},v)=1\},\;N_{j}\leq L.$$

If patient *j* never progressed in the period $\left[t,u_{L}\right]$, as we initially supposed, all $N_{j}$ tests in $\left\{s_{1},\ldots ,s_{N_{j}}\right\}$ will be conducted. However, fewer tests will be performed if the patient did progress at some point $T_{j}^{*}<u_{L}$. We formally define the discrete random variable ${𝒩}_{j}$ denoting the number of performed tests in conjunction with the true progression time $T_{j}^{*}$ as,

$${𝒩}_{j}(S_{j}^{\kappa })=\left\{\begin{array}{ll} 1, & \text{if}\;t<T_{j}^{*}\leq s_{1},\\ 2, & \text{if}\;s_{1}<T_{j}^{*}\leq s_{2},\\ \vdots & \\ N_{j}, & \text{if}\;s_{N_{j}-1}<T_{j}^{*}\leq s_{N_{j}}, \end{array}\right.$$

where $S_{j}^{\kappa }=\{s_{1},\ldots ,s_{N_{j}}\}$ is the schedule of planned future tests. To understand ${𝒩}_{j}(S_{j}^{\kappa })$, consider Figure 3 wherein the schedule contains two planned future tests at future visit times $u_{2}=3.5$ and $u_{4}=5.5$ years. Suppose that when the patient undergoes a real test at $u_{2}=3.5$ years, progression is detected and the patient is removed from surveillance. Then, the total tests performed will be ${𝒩}_{j}(S_{j}^{\kappa })=1$. On the other hand, if progression is detected on a real test at $u_{4}$ then the total tests performed will be ${𝒩}_{j}(S_{j}^{\kappa })=2$. In a real world situation it is not known when a patient will progress and how many of the planned tests will be really conducted. However, we can obtain a personalized estimate of the number of future tests that will get conducted, denoted by the expected value $E\{{𝒩}_{j}(S_{j}^{\kappa })\}$, and defined as,

(4) $$E\{{𝒩}_{j}(S_{j}^{\kappa })\}=\overset{N_{j}}{\underset{n=1}{\sum }}n\times \mathrm{Pr}(s_{n-1}<T_{j}^{*}\leq s_{n}|T_{j}^{*}\leq s_{N_{j}}),\;s_{0}=t,$$

where

$$\mathrm{Pr}(s_{n-1}<T_{j}^{*}\leq s_{n}|T_{j}^{*}\leq s_{N_{j}})=\frac{R_{j}(s_{n}|t,v)-R_{j}(s_{n-1}|t,v)}{R_{j}(s_{N_{j}}|t,v)}.$$

Similarly, we can define the expected time delay in detecting progression, under the assumption that progression occurs before $u_{L}$. Specifically, the random variable time delay is equal to the difference between the time of the test at which progression is observed and the true time of progression $T_{j}^{*}$, and is given by,

$${𝒟}_{j}(S_{j}^{\kappa })=\left\{\begin{array}{ll} s_{1}-T_{j}^{*}, & \text{if}\;t<T_{j}^{*}\leq s_{1},\\ s_{2}-T_{j}^{*}, & \text{if}\;s_{1}<T_{j}^{*}\leq s_{2},\\ \vdots & \\ s_{N_{j}}-T_{j}^{*}, & \text{if}\;s_{N_{j}-1}<T_{j}^{*}\leq s_{N_{j}}, \end{array}\right.$$

The expected time delay in detecting progression is the expected value of ${𝒟}_{j}(S_{j}^{\kappa })$, given by the expression,

(5) $$E\{{𝒟}_{j}(S_{j}^{\kappa })\}=\overset{N_{j}}{\underset{n=1}{\sum }}\{s_{n}-E(T_{j}^{*}|s_{n-1},s_{n},v)\}\times \mathrm{Pr}(s_{n-1}<T_{j}^{*}\leq s_{n}|T_{j}^{*}\leq s_{N}),$$

where $E(T_{j}^{*}|s_{n-1},s_{n},v)$ denotes the conditional expected time of progression for the scenario $s_{n-1}<T_{j}^{*}\leq s_{n}$ and is calculated as the area under the corresponding survival curve,

$$E(T_{j}^{*}|s_{n-1},s_{n},v)=s_{n-1}+\int_{s_{n-1}}^{s_{n}}\mathrm{Pr}\{T_{j}^{*}\geq u|s_{n-1}<T_{j}^{*}\leq s_{n},{𝒴}_{1j}(v),\ldots ,{𝒴}_{Kj}(v),{𝒜}_{n}\}du.$$

The delay calculation shown in (5) can be modified to also handle scenarios wherein a patient has progressed in a certain interval $s_{n-1}<T_{j}^{*}\leq s_{n}$, and the aim is to know if the actual delay is large. The estimated delay in this specific situation is given by:

$$E\{{𝒟}_{j}(S_{j})|s_{n-1}<T_{j}^{*}\leq s_{n}\}=\{s_{n}-E(T_{j}^{*}|s_{n-1},s_{n},v)\}\times \mathrm{Pr}(s_{n-1}<T_{j}^{*}\leq s_{n}|T_{j}^{*}\leq s_{N}).$$

Here, $\mathrm{Pr}(s_{n-1}<T_{j}^{*}\leq s_{n}|T_{j}^{*}\leq s_{N})=1$ because we know that $s_{n-1}<T_{j}^{*}\leq s_{n}$. Thus,

(6) $$E\{{𝒟}_{j}(S_{j})|s_{n-1}<T_{j}^{*}\leq s_{n}\}=s_{n}-E(T_{j}^{*}|s_{n-1},s_{n},v),$$

The personalized schedule in (3), and the corresponding personalized expected number of tests and the expected time delay, all have the advantage of getting updated with newly collected data over follow‐up. Also, the expected number of tests and time delay can be calculated for any schedule, fixed or personalized. Hence, patients/doctors can use them to compare different schedules. Although, a fair comparison of time delays between different schedules for the same patient, requires a compulsory test at a common horizon time point in all schedules.

### 3.4 How to select the risk threshold κ

The risk threshold κ controls the timing and the total number of invasive tests in the personalized schedule $S_{j}^{\kappa }$. Through the timing and the total number of planned tests, κ also indirectly affects the potential time delay (Figure 1) in detecting progression if a particular schedule is followed. Hence, κ should be chosen while balancing both the number of invasive tests (burden) and the time delay in detecting progression (shorter is beneficial).

**FIGURE 4:**
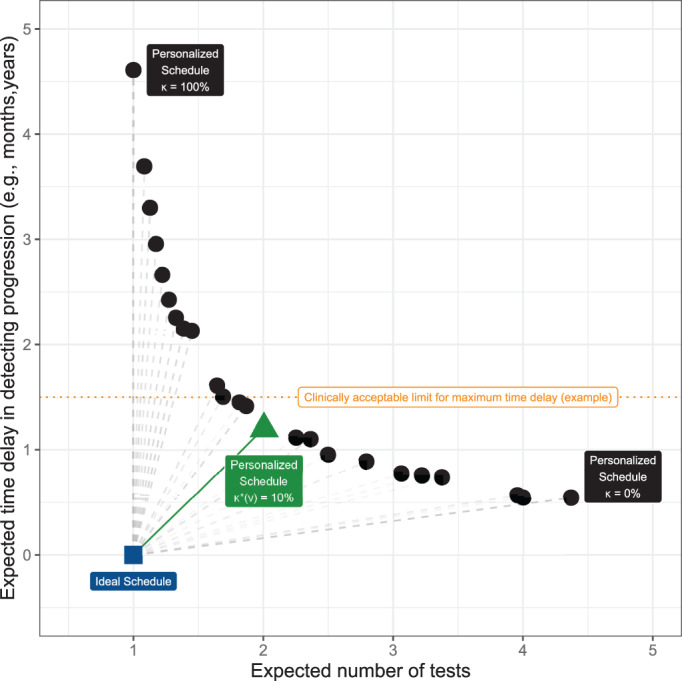
Optimal current‐visit time *v* specific risk threshold $\kappa^{*}(v)$ **obtained using** (7) for the patient shown in Figure 3. Ideal schedule of tests: point (1,0) shown as a blue square. It plans exactly one invasive test at the true time of progression $T_{j}^{*}$ of a patient. Hence, the time delay in detecting progression is zero. Various personalized schedules based on a grid of thresholds κ∈[0,1] are shown with black circles. Higher thresholds lead to fewer tests, but also higher expected time delay. The personalized schedule based on $\kappa^{*}(v)=9.5\%$ threshold (green triangle) has the least Euclidean distance (solid green line) to the ideal schedule. It is also possible to optimize the least distance under a certain clinically acceptable limit on the time delay (dotted horizontal orange line)

To facilitate the choice of κ in practice, following our developments in the previous section, we translate the different choices for threshold κ into the expected number of tests and time delay. In particular, for a patient *j* having data available up to his current visit time *v*, we can construct a bi‐dimensional Euclidean space of his expected total number of tests and expected time delay in detecting progression, for different personalized test schedules obtained by varying the threshold κ∈[0,1]. To illustrate this Euclidean space, we use the example patient shown in Figure 3. For this patient, using (2) we obtained 200 schedules corresponding to 200 risk thresholds between 0% and 100% separated by every 0.5%. For each such schedule, we obtained the personalized expected number of tests and personalized expected delay using (4) and (5), respectively, and plotted them in two dimensions in Figure 4.

The ideal schedule (blue rectangle in Figure 4) for *j*‐th patient is the one in which only one test is conducted, at exactly the true time of progression $T_{j}^{*}$. In other words, the time delay will be zero. If we weigh the expected number of tests and time delay as equally important, then we can select as the optimal threshold at current visit time *v*, the threshold $\kappa^{*}(v)$ which minimizes the Euclidean distance (dashed gray lines connecting the black circles and blue rectangles in Figure 4) between the ideal schedule, that is, point (1, 0) and the set of points representing the different personalized schedules $S_{j}^{\kappa }$ corresponding to various κ∈[0,1], that is,

(7) $$\kappa^{*}(v)=\underset{0\leq \kappa \leq 1}{\text{arg min}}\sqrt{{\left[E\{{𝒩}_{j}(S_{j}^{\kappa })\}-1\right]}^{2}+{\left[E\{{𝒟}_{j}(S^{\kappa })\}-0\right]}^{2}}.$$

In certain scenarios, patients/doctors may be apprehensive about undergoing more than a maximum expected number of future tests, or having an expected time delay higher than certain months. For such purposes, the Euclidean distance in  (7) can be optimized under constraints on the expected number of tests or expected time delay (Figure 4). Doing so alleviates two problems, namely, that the time delay and the number of tests have different units of measurement, and that in (7) they are weighted equally.^26^

We considered shorter delays in detecting progression as the benefit of repeated tests. However, in the literature, decision‐theoretic measures such as quality‐adjusted life‐years/expectancy (QALY/QALE) gained^27^ have also been used to quantify the benefit of testing. Optimizing (7) with QALE needs, setting the optimal point in a Euclidean space with QALE as a dimension, and obtaining expected QALEs for different schedules. For estimating the expected QALE in a personalized manner, a mathematical definition of QALE in terms of time delay ${𝒟}_{j}$ in detecting progression^28^ is required.

## 4 APPLICATION OF PERSONALIZED SCHEDULES IN PROSTATE CANCER SURVEILLANCE

We next demonstrate personalized schedules for scheduling biopsies in prostate cancer active surveillance. To this end, we use results from a joint model fitted to the PRIAS dataset introduced in Section 1. The model definition (Supplementary Material Section B) utilized a linear mixed submodel for biannually measured PSA (continuous: log‐transformed from ng/mL), and a logistic mixed submodel for biannually measured DRE (binary: tumor palpable or not). In the survival submodel, fitted PSA value, fitted instantaneous PSA velocity (defined in Section 2.2), and log‐odds of having a DRE indicating a palpable tumor, were included as time‐dependent predictors. The model parameters were estimated under the Bayesian framework using the R package **JMbayes**,^29^ and are presented in Supplementary Material Section B. We next briefly present the key results relevant for personalized scheduling.

First, the cause‐specific cumulative‐risk of cancer progression at the maximum study period of 10 years was 50% (Supplementary Material Figure 1). This indicates that many patients may not require all of the yearly biopsies they are usually prescribed. Since personalized schedules are risk‐based, their overall performance is dependent on the predictive accuracy and discrimination capacity of the fitted model. In this regard, the model had a moderate time‐dependent area under the receiver operating characteristic curve or AUC^30^ over the follow‐up period (between 0.61 and 0.68). The time‐dependent mean absolute prediction error or MAPE^30^ was moderate to large (between 0.08 and 0.24) and decreased rapidly after year one of the follow‐up. Thus, personalized schedules based on this model may work better after year one with more follow‐up data. Details on AUC and MAPE are provided in Supplementary Material Section B.

### 4.1 Personalized biopsy schedules for a demonstration patient

**FIGURE 5:**
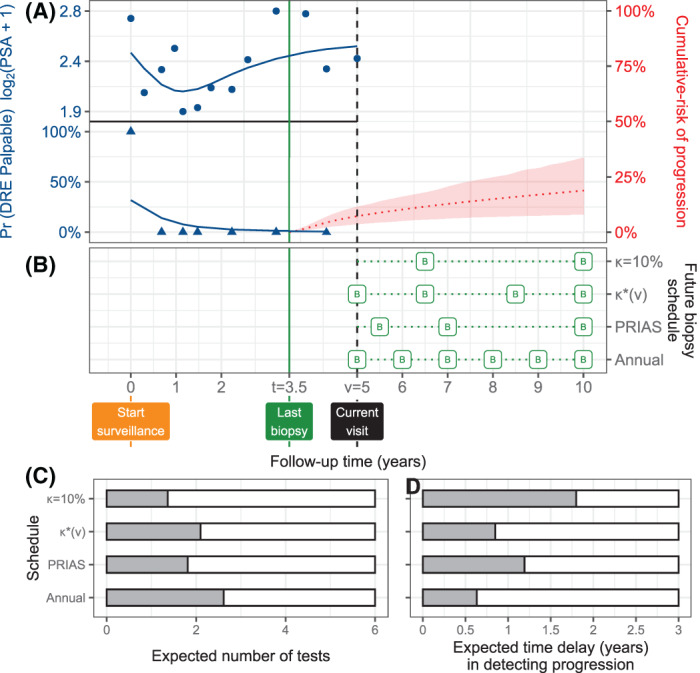
Personalized schedules for a demonstration prostate cancer patient. (A) Time of current visit: v=5 years (black dashed line). Time of last negative biopsy: t=3.5 years (vertical green solid line). Longitudinal data: $\log_{2}(\text{PSA}+1)$ transformed PSA (observed: blue dots, fitted: solid blue line), and binary DRE (observed: blue triangles, fitted probability: solid blue line). Cumulative‐risk profile: dotted red line (95% credible interval shaded). (B) Biopsy indicated with a “B”, and κ=10% and $\kappa^{*}(v)$ are personalized biopsy schedules using a risk threshold of 10%, and a visit time *v* specific automatically chosen threshold (7), respectively. PRIAS and Annual denote the PRIAS biopsy schedule (Section 4.1) and annual biopsy schedule. (C,D) For all schedules we calculate the expected number of tests and expected time delay in detecting progression if the patient progresses before year ten. Since a recommended minimum gap of 1 year is maintained between biopsies, maximum possible number of tests are six. A delay in detecting progression of up to 3 years may not lead to adverse outcomes^31^

We utilized the joint model fitted to the PRIAS dataset to schedule biopsies in a demonstration prostate cancer patient shown in Figure 5. His last negative biopsy was t=3.5 years, and the time of the current visit was v=5 years. We made biopsy decisions over his future visits for PSA measurement $U=\{u_{1}=5,u_{2}=5.5,\ldots ,u_{L}=10\}$ years using four different schedules. Two of the fixed schedules are the annual biopsy schedule and the PRIAS schedule. The PRIAS schedule has compulsory biopsies at years one, four, seven, and ten of follow‐up, and additional annual biopsies if PSA doubling‐time^2^ is high. The remaining two schedules are personalized, namely, with a fixed threshold κ=10% risk and an automatically chosen current visit time *v* specific risk $\kappa^{*}(v)$. To obtain the schedule $\kappa^{*}(v)$, we created 200 risk‐based schedules with 200 different thresholds separated by a 0.5% gap between a 0 and 100% risk window. For each of these risk‐based schedules we obtained the expected number of tests and expected delay in detecting progression using (4) and (5). Subsequently, using (7) we found that $\kappa^{*}(v)=5\%$. That is, the schedule that optimized the Euclidean distance to the ideal schedule (blue rectangle in Figure 4) was a risk‐based schedule that planned a biopsy whenever our demonstration patient's cumulative‐risk of progression was more than 5% (see Figure 3 for planning illustration). Compared to the PRIAS schedule, the $\kappa^{*}(v)$ based schedule leads to an expected 0.2 tests more while reducing the delay by 0.2 years. It is interesting to note that the schedule is based on 10% threshold κ=10% planned biopsies with a very large gap of 3.5 years between the two biopsies, one at 6.5 years and another 10 years. This is due to the fact the cumulative‐risk of progression of the demonstration patient increases 3% yearly on average, up to 19% at the maximum study period of 10 years. Hence, the patient progresses slowly. The delay should be interpreted in a clinical context as well. For example, in prostate cancer active surveillance, a time delay in detecting progression up to 3 years may not lead to adverse downstream outcomes if the time of progression is after year one of follow‐up.^31^ Since the demonstration patient's time of last negative biopsy t=3.5 is after year one of follow‐up, it can be said that even the κ=10% based schedule is safe. Besides we can see that risk‐based personalized approaches also plan fewer biopsies than the annual schedule (Figure 5B), offering a suitable alternative to the annual schedule. Although, with the expected number of tests and expected time delay in detecting progression available for both personalized and fixed schedules, patients and their doctors can evaluate all schedules and make a shared decision.

## 5 SIMULATION STUDY

Although we evaluated personalized schedules for a demonstration patient, we also intend to analyze and compare personalized and fixed schedules in a full cohort. Our criteria for comparison of schedules are the total number of invasive tests planned (burden), and the actual time delay in detecting progression (shorter is beneficial) for each schedule. Due to the periodical nature of schedules, the actual time delay in detecting progression cannot be observed in real‐world surveillance. Hence, instead, we compare personalized vs fixed schedules via an extensive simulated randomized clinical trial in which each hypothetical patient undergoes each schedule. To keep our simulation study realistic, we employ the prostate cancer active surveillance scenario. Specifically, our simulated population is generated using the joint model fitted to the PRIAS cohort (Supplementary Material Section B).

### 5.1 Simulation setup

From the simulation population, we first sample 500 datasets, each representing a hypothetical prostate cancer surveillance program with 1000 patients in it. We sample longitudinal DRE and PSA measurements biannually (PRIAS protocol) for each of the 500×1000 patients and then generate a true cancer progression time for them. We split each dataset into training (750 patients) and test (250 patients) parts, and generate a random and noninformative censoring time for the training patients. All training and test patients also observe Type‐I censoring at year ten of follow‐up (current study period of PRIAS). We next fit a joint model of the same specification as the model fitted to PRIAS (Supplementary Material Section B), to each of the 500 training datasets and retrieve MCMC samples from the 500 sets of the posterior distribution of the parameters. In each of the 500 hypothetical surveillance programs, we utilize the corresponding fitted joint models to obtain the cumulative‐risk of progression in each of the 500×250 test patients. These cumulative‐risk profiles are further used to create personalized biopsy schedules for the test patients.

For each test patient, we conduct hypothetical biopsies using two fixed (PRIAS and annual schedule) and three personalized biopsy schedules. Personalized schedules are based on, a fixed risk threshold κ=10%, an optimal current visit time *v* specific threshold $\kappa^{*}(v)$ chosen via (7), and an optimal threshold obtained under the constraint that expected time delay in detecting progression is less than 0.75 years (9 months), denoted $\kappa^{*}\{v|E(𝒟)\leq 0.75\}$. The choice of 0.75 years delay constraint is arbitrary and is only used to illustrate that applying the constraint limits the average delay at 0.75 years. Successive personalized biopsy decisions are made only on the standard PSA follow‐up visits, utilizing clinical data accumulated only until the corresponding current visit time (2). We maintain a minimum recommended gap of 1 year between consecutive prostate biopsies^2^ as well. Biopsies are conducted until progression is detected, or the maximum follow‐up period at year ten (horizon) is reached. The actual time delay in detecting progression is equal to the difference in time at which progression is detected and the actual (simulated) time of progression of a patient.

### 5.2 Simulation results

**FIGURE 6:**
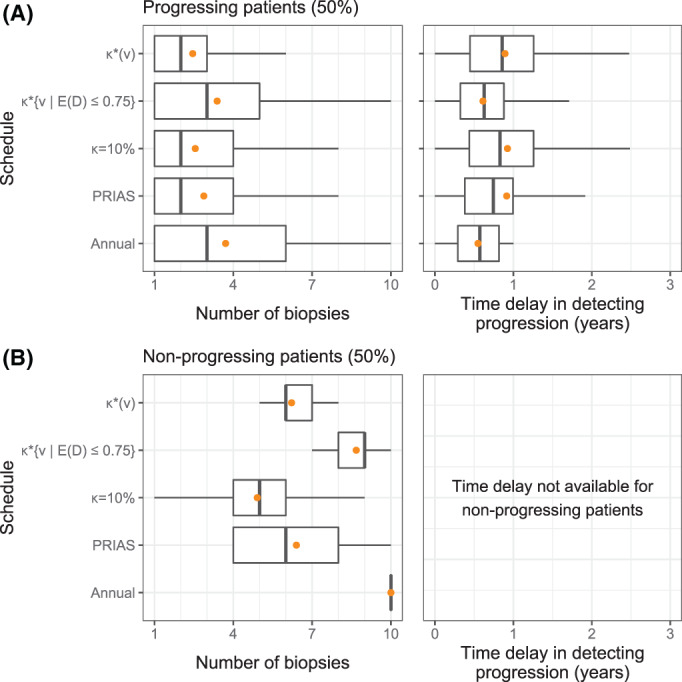
Number of biopsies and the time delay in detecting cancer progression for various biopsy schedules obtained via a simulation study. **Mean** is indicated by the orange circle. Time delay (years) is calculated as (time of positive biopsy—the actual simulated time of cancer progression). Biopsies are conducted until cancer progression is detected. (A) simulated patients who obtained cancer progression in the 10 year study period (progressing). (B) Simulated patients who did not obtain cancer progression in the 10 year study period (nonprogressing). Types of schedules: κ=10% and $\kappa^{*}(v)$ schedule a biopsy if the cumulative‐risk of cancer progression at the current visit time *v* is more than 10%, and an automatically chosen threshold (7), respectively. Schedule $\kappa^{*}\{v|E(𝒟)\leq 0.75\}$ is similar to $\kappa^{*}(v)$ except that the Euclidean distance in (7) is minimized under the constraint that expected delay in detecting progression is at most 9 months (0.75 years). Annual corresponds to a schedule of yearly biopsies, and PRIAS corresponds to biopsies as per PRIAS protocol (Section 4)

In the simulation study, nearly 50% of the patients observed progression during 10 year study period (*progressing*) and 50% did not (*nonprogressing*). While we can calculate the total number of biopsies scheduled in all 500×250 test patients, the actual time delay in detecting progression is available only for progressing patients. Hence, we show the simulation results separately for progressing and nonprogressing patients (Figure 6).

Before discussing delay in detecting progression (Figure 6A), we note that mean delay up to 1.7 years in all patients,^32^ and up to 3 years in patients who progress after year one of follow‐up,^31^ may not increase risks of adverse outcomes later. In this regard, the annual biopsies guarantee a maximum delay of 1 year in all patients. However, they also schedule the highest number of biopsies (Median 3, Inter‐quartile range or IQR: 1‐6). Much fewer biopsies are planned by the PRIAS schedule (Median 2, IQR: 1‐4), but it also has a higher time delay (Median 0.74, IQR: 0.38‐1.00 years). The personalized schedule based on optimal risk threshold $\kappa^{*}(v)$ schedules fewer biopsies than PRIAS and has a delay (Median 0.86, IQR: 0.46‐1.26 years) slightly higher than PRIAS. The expected delay for risk threshold optimized with a constraint on expected delay $\kappa^{*}\{v|E(D)\leq 0.75\}$ is equal to 0.61 years, that is, the constraint works as expected.

The simulated nonprogressing patients (Figure 6B) gained the most with personalized schedules. The annual schedule plans 10 (unnecessary) biopsies for each such patient, and the PRIAS schedule plans a median of 6 (IQR: 4‐8) biopsies. In contrast, the personalized schedule based on optimized risk threshold $\kappa^{*}(v)$ plans fewer biopsies consistently (Median 6, IQR: 6‐7). The 10% threshold based schedule plans even fewer biopsies (Median 5, IQR: 4‐6).

## 6 DISCUSSION

In this article, we presented a methodology to create personalized schedules for burdensome diagnostic *tests* used to detect disease *progression* in early‐stage chronic noncommunicable disease *surveillance*. For this purpose, we utilized joint models for time‐to‐event and longitudinal data. Our approach first combines a patient's clinical data (eg, longitudinal biomarkers) and previous invasive test results to estimate patient‐specific cumulative‐risk of disease progression over their current and future follow‐up visits. We then plan future invasive tests whenever this cumulative‐risk of progression is predicted to be above a certain threshold. We select the risk threshold automatically in a personalized manner, by optimizing a utility function of the patient‐specific consequences of choosing a particular risk threshold based schedule. These consequences are, namely, the number of invasive tests (burden) planned in a schedule, and the expected time delay in detection of progression (shorter is beneficial) if the patient progresses. Last, we calculate this expected time delay in a personalized manner for both personalized and fixed schedules to assist patients/doctors in making a more informed and shared decision of choosing a test schedule.

Using joint models gives us certain advantages. First, since joint models employ random‐effects, the corresponding risk‐based schedules are inherently personalized. Second, to predict this patient‐specific cumulative‐risk of progression, joint models utilize all observed longitudinal measurements of a patient. Also, the continuous longitudinal outcomes are not discretized, which is commonly a case in Markov Decision Process and flowchart‐based test schedules. Third, personalized schedules update automatically with more patient data over follow‐up. Fourth, we calculated the expected number of tests (burden) and expected time delay in detecting progression (shorter is beneficial) in a patient‐specific manner. Using our methodology, these can be calculated for both personalized and fixed schedules. Thus, patients/doctors can compare risk‐based and fixed schedules and make a shared decision of a test schedule according to their preferences for the expected burden‐benefit ratio. While based on these arguments we propose the use of joint models for predicting risks, the methodology in Section 3 can be used with any other model that provides risk estimates for progression. Last, although this work concerns invasive test schedules in disease surveillance, the methodology is generic for use under a screening setting as well.

Personalized schedules that we proposed require a risk threshold. We optimized the threshold choice using a generic utility function based on the expected number of biopsies and time delay in detecting progression. We used only these two measures because they are easy to interpret but simultaneously critical for deciding the timing of invasive tests. Also, the time delay in detecting progression is an easily‐quantifiable surrogate for the window of opportunity for curative treatment and additional benefits of observing progression early. Practitioners may extend/modify our utility function by adding to/replacing time delay with commonly used decision‐theoretic measures such as quality‐adjusted life‐years/expectancy (QALY/QALE). While a key aspect of our schedules is that they automatically update over time, this also means that the testing decisions beyond the current visit times are less meaningful without the most updated information collected in future clinical visits. On the other hand, generating a complete planned schedule allows patients to have a more informed idea of their disease situation, and what awaits them. Specifically, knowing a future schedule and the consequences (expected time delay in detecting progression and expected number of tests) of following the future schedule can also assist both doctors and patients make better shared decisions of tests. Specifically, if the subject finds a proposed personalized schedule burdensome or too lax, they can also compare it with the existing schedules in a quantitative manner. In addition, from a healthcare/medical center perspective, projected schedules for patients are informative for better healthcare resource planning and demand redistribution. This issue has especially been of practical relevance in the current COVID‐19 pandemic. Such advantages are not available in methodologies that make a single test decision.

We evaluated personalized schedules in a full cohort via a realistic simulation of a randomized clinical trial for prostate cancer surveillance patients. We observed that personalized schedules reduced many unnecessary biopsies for nonprogressing patients compared to the widely used annual schedule. This happened at the cost of simultaneously having a slightly longer time delay in detecting progression. Although, this delay should still be safe because it was almost equal to the delay of the world's largest prostate cancer active surveillance program PRIAS's schedule. The simulation study results are by no means the performance‐limit of the personalized schedules. On the contrary, our approach will become more personalized as the predictive ability of the marker(s) improves. Thus models with higher predictive accuracy and discrimination capacity than the PRIAS based demonstration model may lead to an even better balance between the number of tests and the time delay in detecting progression. As for the practical usability of the PRIAS based model in prostate cancer surveillance, the model needs external validation and improvements in its predictive performance. Despite that, we expect this model's overall impact to be positive. There are two reasons for this. First, the risk of adverse outcomes because of personalized schedules is quite low because of the low rate of metastases and prostate cancer specific mortality in prostate cancer patients.^2^ Second, studies^31^, ^32^ have suggested that after the confirmatory biopsy at year one of follow‐up, biopsies may be done as infrequently as every 2 to 3 years, with limited adverse consequences. In other words, longer delays in detecting progression may be acceptable after the first negative biopsy.

There are certain limitations to this work. First, in practice, most cohorts have a limited study period. Hence, the cumulative‐risk profiles of patients and resulting personalized schedules can only be created up to the maximum study period. For this problem, the risk prediction model should be updated with more follow‐up data over time. The proposed joint model assumed all events other than progression to be noninformative censoring, and consequently the cumulative‐risk of progression is over‐estimated. Better estimates may be obtained by using models that account for competing risks. The detection of progression is susceptible to inter‐observer variation, for example, pathologists may grade the same biopsy differently. Progression is sometimes obscured due to sampling error, for example, biopsy results vary based on location and number of biopsy cores. Although models that account for inter‐observer variation^33^ and sampling error^34^ will provide better risk estimates, the methodology for obtained personalized schedules can remain the same.

## CONFLICT OF INTEREST

The authors declare no potential conflict of interests.

## AUTHOR CONTRIBUTIONS

**Anirudh Tomer** had full access to all the data in the study and takes responsibility for the integrity of the data and the accuracy of the data analysis. **Anirudh Tomer, Ewout W. Steyerberg, Dimitris Rizopoulos**: Concept and ideas. **Anirudh Tomer, Daan Nieboer, Monique J. Roobol**: Acquisition of data. **Anirudh Tomer, Daan Nieboer, Dimitris Rizopoulos**: Analysis and interpretation of data. **Anirudh Tomer, Dimitris Rizopoulos**: Drafting of the manuscript. **Anirudh Tomer, Daan Nieboer, Ewout W. Steyerberg, Dimitris Rizopoulos**: Critical revision of the manuscript for important intellectual content. **Anirudh Tomer, Daan Nieboer, Monique J. Roobol, Ewout W. Steyerberg, Dimitris Rizopoulos**: Statistical analyses. **Monique J. Roobol, Ewout W. Steyerberg, Dimitris Rizopoulos**: Obtaining funding. **Daan Nieboer**: Administrative, technical or material support. **Dimitris Rizopoulos**: Supervision.

## Supporting information


**Data S1** Supplementary Material

## Data Availability

This simulation study utilized results from a statistical model fitted to the PRIAS dataset. The PRIAS database is not openly accessible. However, access to the database can be requested on the basis of a study proposal approved by the PRIAS steering committee. The website of the PRIAS program is https://www.prias‐project.org. Instructions for generating a synthetic dataset are provided in the README file along with the source code.

## References

[1] WHO . Global status report on noncommunicable diseases 2014, Report No. WHO/NMH/NVI/15.1, World Health Organization; 2014.

[2] Bokhorst LP , Alberts AR , Rannikko A , et al. Compliance rates with the Prostate Cancer Research International Active Surveillance (PRIAS) protocol and disease reclassification in noncompliers. Eur Urol. 2015;68(5):814‐821. 26138043 10.1016/j.eururo.2015.06.012

[3] Weusten B , Bisschops R , Coron E , et al. Endoscopic management of Barrett's esophagus: European Society of Gastrointestinal Endoscopy (ESGE) position statement. Endoscopy. 2017;49(02):191‐198. 28122386 10.1055/s-0042-122140

[4] Krist AH , Jones RM , Woolf SH , et al. Timing of repeat colonoscopy: disparity between guidelines and endoscopists' recommendation. Am J Prevent Med. 2007;33(6):471‐478. 10.1016/j.amepre.2007.07.039 18022063

[5] McWilliams TJ , Williams TJ , Whitford HM , Snell GI . Surveillance bronchoscopy in lung transplant recipients: risk versus benefit. J Heart Lung Transpl. 2008;27(11):1203‐1209. 10.1016/j.healun.2008.08.004 18971092

[6] Loeb S , Vellekoop A , Ahmed HU , et al. Systematic review of complications of prostate biopsy. Eur Urol. 2013;64(6):876‐892. 23787356 10.1016/j.eururo.2013.05.049

[7] Le Clercq C , Winkens B , Bakker C , et al. Metachronous colorectal cancers result from missed lesions and non‐compliance with surveillance. Gastrointest Endosc. 2015;82(2):325‐333.e2. 25843613 10.1016/j.gie.2014.12.052

[8] Alagoz O , Ayer T , Erenay FS . Operations Research Models for Cancer Screening. Wiley Encyclopedia of Operations Research and Management Science. Hoboken, NJ: John Wiley & Sons; 2011.

[9] Steimle LN , Denton BT . Markov Decision Processes for Screening and Treatment of Chronic Diseases. Berlin, Germany: Springer International Publishing; 2017.

[10] Vickers AJ , Elkin EB . Decision curve analysis: a novel method for evaluating prediction models. Med Decis Making. 2006;26(6):565‐574. 17099194 10.1177/0272989X06295361 PMC2577036

[11] Tomer A , Nieboer D , Roobol MJ , Steyerberg EW , Rizopoulos D . Personalized schedules for surveillance of low‐risk prostate cancer patients. Biometrics. 2019;75(1):153‐162. 30039528 10.1111/biom.12940 PMC7380003

[12] Bebu I , Lachin JM . Optimal screening schedules for disease progression with application to diabetic retinopathy. Biostatistics. 2017;19(1):1‐13. 10.1093/biostatistics/kxx009 PMC6075595 28430872

[13] Rizopoulos D , Taylor JM , Van Rosmalen J , Steyerberg EW , Takkenberg JJ . Personalized screening intervals for biomarkers using joint models for longitudinal and survival data. Biostatistics. 2015;17(1):149‐164. 26319700 10.1093/biostatistics/kxv031 PMC4679074

[14] Tomer A , Rizopoulos D , Nieboer D , Drost FJ , Roobol MJ , Steyerberg EW . Personalized decision making for biopsies in prostate cancer active surveillance programs. Med Decis Making. 2019;39(5):499‐508. 31319751 10.1177/0272989X19861963 PMC6791024

[15] Wang Y , Zhao YQ , Zheng Y . Learning‐based biomarker‐assisted rules for optimized clinical benefit under a risk‐constraint. Biometrics. 2020;76(3):853‐862. 31833561 10.1111/biom.13199 PMC7292743

[16] Tsiatis AA , Davidian M . Joint modeling of longitudinal and time‐to‐event data: an overview. Stat Sin. 2004;14(3):809‐834.

[17] Rizopoulos D . Joint Models for Longitudinal and Time‐to‐Event Data: With Applications in R. Boca Raton, FL: CRC Press; 2012.

[18] McCulloch CE , Neuhaus JM . Generalized linear mixed models. Encyclopedia of Biostatistics. Vol 4; Hoboken, NJ: John Wiley & Sons; 2005.

[19] Loeb S , Bjurlin MA , Nicholson J , et al. Overdiagnosis and overtreatment of prostate cancer. Eur Urol. 2014;65(6):1046‐1055. 24439788 10.1016/j.eururo.2013.12.062 PMC4113338

[20] Epstein JI , Egevad L , Amin MB , Delahunt B , Srigley JR , Humphrey PA . The 2014 International Society of Urological Pathology (ISUP) consensus conference on Gleason grading of prostatic carcinoma. Am J Surg Pathol. 2016;40(2):244‐252. 26492179 10.1097/PAS.0000000000000530

[21] Loeb S , Carter HB , Schwartz M , Fagerlin A , Braithwaite RS , Lepor H . Heterogeneity in active surveillance protocols worldwide. Rev Urol. 2014;16(4):202‐203. 25548550 PMC4274180

[22] Torre LA , Bray F , Siegel RL , Ferlay J , Lortet‐Tieulent J , Jemal A . Global cancer statistics, 2012. CA Cancer J Clin. 2015;65(2):87‐108. 25651787 10.3322/caac.21262

[23] Brown ER . Assessing the association between trends in a biomarker and risk of event with an application in pediatric HIV/AIDS. Ann Appl Stat. 2009;3(3):1163‐1182. 20802852 10.1214/09-aoas251 PMC2928653

[24] Taylor JM , Park Y , Ankerst DP , et al. Real‐time individual predictions of prostate cancer recurrence using joint models. Biometrics. 2013;69(1):206‐213. 23379600 10.1111/j.1541-0420.2012.01823.x PMC3622120

[25] Eilers PH , Marx BD . Flexible smoothing with B‐splines and penalties. Stat Sci. 1996;11(2):89‐121.

[26] Cook RD , Wong WK . On the equivalence of constrained and compound optimal designs. J Am Stat Assoc. 1994;89(426):687‐692.

[27] Sassi F . Calculating QALYs, comparing QALY and DALY calculations. Health Policy Plan. 2006;21(5):402‐408. 16877455 10.1093/heapol/czl018

[28] de Carvalho TM , Heijnsdijk EA , de Koning HJ . When should active surveillance for prostate cancer stop if no progression is detected? Prostate. 2017;77(9):962‐969. 28419541 10.1002/pros.23352

[29] Rizopoulos D , The R . Package JMbayes for fitting joint models for longitudinal and time‐to‐event data using MCMC. J Stat Softw. 2016;72(7):1‐46.

[30] Rizopoulos D , Molenberghs G , Lesaffre EM . Dynamic predictions with time‐dependent covariates in survival analysis using joint modeling and landmarking. Biometr J. 2017;59(6):1261‐1276. 10.1002/bimj.201600238 28792080

[31] de Carvalho TM , Heijnsdijk EA , de Koning HJ . Estimating the risks and benefits of active surveillance protocols for prostate cancer: a microsimulation study. BJU Int. 2017;119(4):560‐566. 27222299 10.1111/bju.13542 PMC5859305

[32] Inoue LY , Lin DW , Newcomb LF , et al. Comparative analysis of biopsy upgrading in four prostate cancer active surveillance cohorts. Ann Internal Med. 2018;168(1):1‐9. 29181514 10.7326/M17-0548 PMC5752581

[33] Balasubramanian R , Lagakos SW . Estimation of a failure time distribution based on imperfect diagnostic tests. Biometrika. 2003;90(1):171‐182.

[34] Coley RY , Zeger SL , Mamawala M , Pienta KJ , Carter HB . Prediction of the pathologic Gleason score to inform a personalized management program for prostate cancer. Eur Urol. 2017;72(1):135‐141. 27523594 10.1016/j.eururo.2016.08.005

