# Supplementary material for: Shared decision making of burdensome surveillance tests using personalized schedules and their burden and benefit
Source: Stat Med. 2022 Feb 10;41(12):2115–31. doi: 10.1002/sim.9347 (PMC9305929; doi:10.1002/sim.9347)
Supplement: Supplementary file 1 — Data S1 Supplementary Material [file SIM-41-2115-s001.pdf]

Supplementary Materials for 'Shared Decision Making of Burdensome Surveillance Tests Using Personalized Schedules and Their Burden and Benefit'

Anirudh Tomer*[1], Daan Nieboer[2], Monique J. Roobol[3], Ewout W. Steyerberg[2,4], and Dimitris Rizopoulos[1]

[1]Department of Biostatistics, Erasmus University Medical Center, the Netherlands
[2]Department of Public Health, Erasmus University Medical Center, the Netherlands
[3]Department of Urology, Erasmus University Medical Center, the Netherlands
[4]Department of Biomedical Data Sciences, Leiden University Medical Center, the Netherlands

October 28, 2021

*Corresponding author: a.tomer@erasmusmc.nl
The authors gratefully acknowledge Nederlandse Organisatie voor Wetenschappelijk Onderzoek VIDI grant nr. 016.146.301, and Erasmus University Medical Center funding.

A Joint Model for Time-to-Progression and Longitudinal Outcomes

Let T_i^* denote the true time of disease progression for the i-th patient. Progression is always interval censored $l_i < T_i^* \leq r_i$. Here, r_i and l_i denote the time of the last and second last invasive tests, respectively, when patients progress. In non-progressing patients, l_i denotes the time of the last test and $r_i = \infty$. Assuming K types of longitudinal outcomes, let $\boldsymbol{y}_{ki}$ denote the $n_{ki} \times 1$ longitudinal response vector of the k-th outcome, $k \in \{1, \ldots, K\}$. The observed data of all n patients is given by $\mathcal{A}_n = \{l_i, r_i, \boldsymbol{y}_{1i}, \ldots \boldsymbol{y}_{Ki}; i = 1, \ldots, n\}$.

A.1 Longitudinal Sub-process

To model multiple longitudinal outcomes in a unified framework, a joint model employs individual generalized linear mixed sub-models [1]. Specifically, the conditional distribution of the k-th outcome $\boldsymbol{y}_{ki}$ given a vector of patient-specific random effects $\boldsymbol{b}_{ki}$ is assumed to belong to the exponential family, with linear predictor given by,

$$g_k\big[E\{y_{ki}(t) \mid \boldsymbol{b}_{ki}\}\big] = m_{ki}(t) = \boldsymbol{x}_{ki}^\top(t)\boldsymbol{\beta}_k + \boldsymbol{z}_{ki}^\top(t)\boldsymbol{b}_{ki},$$

where $g_k(\cdot)$ denotes a known one-to-one monotonic link function, $y_{ki}(t)$ is the value of the k-th longitudinal outcome for the i-th patient at time t, and $\boldsymbol{x}_{ki}(t)$ and $\boldsymbol{z}_{ki}(t)$ are the time-dependent design vectors for the fixed $\boldsymbol{\beta}_k$ and random effects $\boldsymbol{b}_{ki}$, respectively. To model the correlation between different longitudinal outcomes, we link their corresponding random effects. Specifically, we assume that the vector of random effects $\boldsymbol{b}_i = (\boldsymbol{b}_{1i}^\top, \ldots, \boldsymbol{b}_{Ki}^\top)^\top$ follows a multivariate normal distribution with mean zero and variance-covariance matrix W.

A.2 Survival Sub-process

In the survival sub-process, the hazard of progression $h_i(t)$ at a time t is assumed to depend on a function of patient and outcome-specific linear predictors $m_{ki}(t)$ and/or the random effects,

$$h_i\big\{t \mid \mathcal{M}_i(t), \boldsymbol{w}_i(t)\big\} = h_0(t) \exp\Big[\boldsymbol{\gamma}^\top \boldsymbol{w}_i(t) + \sum_{k=1}^{K} f_k\big\{\mathcal{M}_{ki}(t), \boldsymbol{w}_i(t), \boldsymbol{b}_{ki}, \boldsymbol{\alpha}_k\big\}\Big], \quad t > 0,$$

where $h_0(\cdot)$ denotes the baseline hazard, $\mathcal{M}_{ki}(t) = \{m_{ki}(s) \mid 0 \leq s < t\}$ is the history of the k-th longitudinal process up to t, and $\boldsymbol{w}_i(t)$ is a vector of exogenous, possibly time-varying covariates with regression coefficients $\boldsymbol{\gamma}$. Functions $f_k(\cdot)$, parameterized by vector of coefficients $\boldsymbol{\alpha_k}$, specify the features of each longitudinal outcome that are included in the linear predictor of the relative-risk model [2, 3, 4]. Some examples, motivated by the literature (subscripts k dropped for brevity), are,

$$\begin{cases} f\big\{\mathcal{M}_i(t), \boldsymbol{w}_i(t), \boldsymbol{b}_i, \boldsymbol{\alpha}\big\} = \alpha m_i(t), \\ f\big\{\mathcal{M}_i(t), \boldsymbol{w}_i(t), \boldsymbol{b}_i, \boldsymbol{\alpha}\big\} = \alpha_1 m_i(t) + \alpha_2 m_i'(t), \quad \text{with } m_i'(t) = \frac{\mathrm{d}m_i(t)}{\mathrm{d}t}. \end{cases}$$

These formulations of $f(\cdot)$ postulate that the hazard of progression at time t may be associated with the underlying level $m_i(t)$ of the longitudinal outcome at t, or with both the level and velocity $m_i'(t)$ (e.g., PSA value and velocity in prostate cancer) of the outcome at t. Lastly, $h_0(t)$ is the baseline hazard at time t, and is modeled flexibly using P-splines [5]. More specifically:

$$\log h_0(t) = \gamma_{h_0,0} + \sum_{q=1}^{Q} \gamma_{h_0,q} B_q(t, \boldsymbol{v}),$$

where $B_q(t, \boldsymbol{v})$ denotes the q-th basis function of a B-spline with knots $\boldsymbol{v} = v_1, \ldots, v_Q$ and vector of spline coefficients γ_{h_0}. To avoid choosing the number and position of knots in the

spline, a relatively high number of knots (e.g., 15 to 20) are chosen and the corresponding B-spline regression coefficients γ_{h_0} are penalized using a differences penalty [5].

A.3 Parameter Estimation

We estimate the parameters of the joint model using Markov chain Monte Carlo (MCMC) methods under the Bayesian framework. Let $\boldsymbol{\theta}$ denote the vector of all of the parameters of the joint model. The joint model postulates that given the random effects, the time to progression, and all of the longitudinal measurements taken over time are all mutually independent. Under this assumption the posterior distribution of the parameters is given by:

$$
\begin{aligned}
p(\boldsymbol{\theta}, \boldsymbol{b} \mid \mathcal{D}_n) &\propto \prod_{i=1}^{n} p(l_i, r_i, \boldsymbol{y}_{1i}, \ldots \boldsymbol{y}_{Ki}, \mid \boldsymbol{b}_i, \boldsymbol{\theta}) p(\boldsymbol{b}_i \mid \boldsymbol{\theta}) p(\boldsymbol{\theta}) \\
&\propto \prod_{i=1}^{n} \prod_{k=1}^{K} p(l_i, r_i \mid \boldsymbol{b}_i, \boldsymbol{\theta}) p(\boldsymbol{y}_{ki} \mid \boldsymbol{b}_i, \boldsymbol{\theta}) p(\boldsymbol{b}_i \mid \boldsymbol{\theta}) p(\boldsymbol{\theta}), \\
p(\boldsymbol{b}_i \mid \boldsymbol{\theta}) &= \frac{1}{\sqrt{(2\pi)^{|W|} \det(\boldsymbol{D})}} \exp(\boldsymbol{b}_i^\top \boldsymbol{D}^{-1} \boldsymbol{b}_i),
\end{aligned}
$$

where, the likelihood contribution of the k-th longitudinal outcome vector $\boldsymbol{y}_{ki}$ for the i-th patient, conditional on the random effects is:

$$
p(\boldsymbol{y}_{ki} \mid \boldsymbol{b}_i, \boldsymbol{\theta}) = \prod_{j=1}^{n_{ki}} \exp \left[\frac{y_{kij} \psi_{kij}(\boldsymbol{b}_{ki}) - c_k\{\psi_{kij}(\boldsymbol{b}_{ki})\}}{a_k(\varphi)} - d_k(y_{kij}, \varphi) \right],
$$

where n_{ki} are the total number of longitudinal measurements of type k for patient i. The natural and dispersion parameters of the exponential family are denoted by $\psi_{kij}(\boldsymbol{b}_{ki}$ and φ, respectively. In addition, $c_k(\cdot), a_k(\cdot), d_k(\cdot)$ are known functions specifying the member of the exponential family. The likelihood contribution of the time to progression outcome

is given by:

$$p(l_i, r_i \mid \boldsymbol{b}_i, \boldsymbol{\theta}) = \exp\Big[-\int_0^{l_i} h_i\big\{s \mid \mathcal{M}_i(t), \boldsymbol{w}_i(t)\big\}\text{d}s\Big] - \exp\Big[-\int_0^{r_i} h_i\big\{s \mid \mathcal{M}_i(t), \boldsymbol{w}_i(t)\big\}\text{d}s\Big]. \tag{1}$$

The integral in (1) does not have a closed-form solution, and therefore we use a 15-point Gauss-Kronrod quadrature rule to approximate it.

We use independent normal priors with zero mean and variance 100 for the fixed effect parameters of the longitudinal model. For scale parameters we inverse Gamma priors. For the variance-covariance matrix $\boldsymbol{D}$ of the random effects we take inverse Wishart prior with an identity scale matrix and degrees of freedom equal to the total number of random effects. For the relative risk model's parameters $\boldsymbol{\gamma}$ and the association parameters $\boldsymbol{\alpha}$, we use independent normal priors with zero mean and variance 100. However, when $\boldsymbol{\alpha}$ becomes high dimensional (e.g., when several functional forms are considered per longitudinal outcome), we opt for a global-local ridge-type shrinkage prior, i.e., for the s-th element of $\boldsymbol{\alpha}$ we assume:

$$\alpha_s \sim \mathcal{N}(0, \tau\psi_s), \quad \tau^{-1} \sim \text{Gamma}(0.1, 0.1), \quad \psi_s^{-1} \sim \text{Gamma}(1, 0.01). \tag{2}$$

The global smoothing parameter τ has sufficiently mass near zero to ensure shrinkage, while the local smoothing parameter ψ_s allows individual coefficients to attain large values. Other options of shrinkage or variable-selection priors could be used as well [6]. Finally, the penalized version of the B-spline approximation to the baseline hazard is specified using the following hierarchical prior for γ_{h_0} [7]:

$$p(\gamma_{h_0} \mid \tau_h) \propto \tau_h^{\rho(\boldsymbol{K})/2} \exp\Big(-\frac{\tau_h}{2}\gamma_{h_0}^\top \boldsymbol{K} \gamma_{h_0}\Big) \tag{3}$$

where τ_h is the smoothing parameter that takes a $\text{Gamma}(1, \tau_{h\delta})$ prior distribution, with a hyper-prior $\tau_{h\delta} \sim \text{Gamma}(10^{-3}, 10^{-3})$, which ensures a proper posterior distribution for

γ_{h_0}[8], $\boldsymbol{K} = \Delta_r^\top \Delta_r + 10^{-6}\boldsymbol{I}$, with Δ_r denoting the r-th difference penalty matrix, and $\rho(\boldsymbol{K})$ denotes the rank of $\boldsymbol{K}$.

B Joint Model for the PRIAS Dataset Used in Simulation Study

B.1 Dataset

This work uses a joint model fitted to the PRIAS dataset (Table 1). The PRIAS database is not openly accessible. However, access to the database can be requested on the basis of a study proposal approved by the PRIAS steering committee. The website of the PRIAS program is `www.prias-project.org`. We have presented the PRIAS based model's definition and parameter estimates below.

Table 1: **Summary of the PRIAS dataset**. The primary event of interest is cancer progression (increase in biopsy Gleason grade group from grade group 1 to 2 or higher). Abbreviations: PSA is prostate-specific antigen; DRE is digital rectal examination, with level T1c [9] indicating a clinically inapparent tumor which is not palpable or visible by imaging, whereas tumors with DRE > T1c are palpable; IQR is interquartile range.

**Characteristic**	**Value**
Total patients	7813
progression (primary event)	1134
Treatment	2250
Watchful waiting	334
Lost to follow-up	203
Discontinued on request	46
Death (other)	95
Death (prostate cancer)	2
Total DRE measurements	37326
Total PSA measurements	67578
Total biopsies	15686
Median age at diagnosis (years)	66 (IQR: 61–71)
Median PSA (ng/mL)	5.7 (IQR: 4.1–7.7)
DRE = T1c (%)	34883/37326 (94%)
Median number of PSA per patient	6 (IQR: 4–12)
Median number of DRE per patient	4 (IQR: 2–7)
Median number of biopsies per patient	2 (IQR: 1–2)

B.2 Model Specification

Let T_i^* denote the true progression time of the i-th patient included in PRIAS. Since biopsies are conducted periodically, T_i^* is observed with interval censoring $l_i < T_i^* \leq r_i$. When progression is observed for the patient at his latest biopsy time r_i, then l_i denotes the time of the second latest biopsy. Otherwise, l_i denotes the time of the latest biopsy and $r_i = \infty$. Let $\boldsymbol{y}_{di}$ and $\boldsymbol{y}_{pi}$ denote his observed DRE (digital rectal examination) and PSA (prostate-specific antigen) longitudinal measurements, respectively. The observed data of all n patients is denoted by $\mathcal{A}_n = \{l_i, r_i, \boldsymbol{y}_{di}, \boldsymbol{y}_{pi}; i = 1, \dots, n\}$.

The patient-specific DRE and PSA measurements over time are modeled using a bivariate generalized linear mixed effects sub-model. The sub-model for DRE is given by:

$$\begin{aligned} \text{logit}\big[\Pr\{y_{di}(t) > \text{T1c}\}\big] &= \beta_{0d} + b_{0di} + (\beta_{1d} + b_{1di})t \\ &\quad + \beta_{2d}(\text{Age}_i - 65) + \beta_{3d}(\text{Age}_i - 65)^2 \end{aligned} \tag{4}$$

where, t denotes the follow-up visit time, and Age_i is the age of the i-th patient at the time of inclusion in AS. The fixed effect parameters are denoted by $\{\beta_{0d}, \dots, \beta_{3d}\}$, and $\{b_{0di}, b_{1di}\}$ are the patient specific random effects. With this definition, we assume that the patient-specific log odds of obtaining a DRE measurement larger than T1c [9], i.e., palpable tumor, remain linear over time.

The mixed effects sub-model for PSA is given by:

$$\begin{aligned} \log_2\big\{y_{pi}(t) + 1\big\} &= m_{pi}(t) + \varepsilon_{pi}(t), \\ m_{pi}(t) &= \beta_{0p} + b_{0pi} + \sum_{k=1}^{3}(\beta_{kp} + b_{kpi})B_k(t, \mathcal{K}) \\ &\quad + \beta_{4p}(\text{Age}_i - 65) + \beta_{5p}(\text{Age}_i - 65)^2, \end{aligned} \tag{5}$$

where, $m_{pi}(t)$ denotes the underlying measurement error free value of $\log_2(\text{PSA} + 1)$ transformed [10, 11] measurements at time t. We model it non-linearly over time using B-

splines [12]. To this end, the B-spline basis function $B_k(t, \mathcal{K})$ has two internal knots at $\mathcal{K} = \{0.75, 2.12\}$ years (33.34 and 66.67 percentile of observed follow-up times), and boundary knots at 0 and 6.4 years (95-th percentile of the observed follow-up times). The fixed effect parameters are denoted by $\{\beta_{0p}, \ldots, \beta_{5p}\}$, and $\{b_{0pi}, \ldots, b_{3pi}\}$ are the patient specific random effects. The error $\varepsilon_{pi}(t)$ is assumed to be t-distributed with three degrees of freedom (Appendix B.4) and scale σ, and is independent of the random effects.

To account for the correlation between the DRE and PSA measurements of a patient, their corresponding random effects are linked. Specifically, the complete vector of random effects $\boldsymbol{b}_i = (b_{0di}, b_{1di}, b_{0pi}, \ldots, b_{3pi})^\top$ is assumed to follow a multivariate normal distribution with mean zero and variance-covariance matrix $\boldsymbol{W}$.

To model the impact of DRE and PSA measurements on the risk of progression, the joint model uses a relative risk sub-model. More specifically, the hazard of progression $h_i(t)$ at a time t is given by:

$$\begin{aligned} h_i(t) = h_0(t) \exp\Big(&\gamma_1 (\text{Age}_i - 65) + \gamma_2 (\text{Age}_i - 65)^2 \\ &+ \alpha_{1d} \text{logit}\big[\Pr\{y_{di}(t) > \text{T1c}\}\big] + \alpha_{1p} m_{pi}(t) + \alpha_{2p} \frac{\partial m_{pi}(t)}{\partial t}\Big), \end{aligned} \tag{6}$$

where, γ_1, γ_2 are the parameters for the effect of age. The parameter α_{1d} models the impact of log odds of obtaining a DRE > T1c on the hazard of progression. The impact of PSA on the hazard of progression is modeled in two ways: a) the impact of the error free underlying PSA value $m_{pi}(t)$, and b) the impact of the underlying PSA velocity $\partial m_{pi}(t)/\partial t$. The corresponding parameters are α_{1p} and α_{2p}, respectively. Lastly, $h_0(t)$ is the baseline hazard at time t, and is modeled flexibly using P-splines [5].

B.3 Parameter Estimates

Figure 1 shows the cumulative-risk of progression over the follow-up period. The posterior parameter estimates for the PRIAS based joint model are given in Table 4 (longitudinal sub-model for DRE outcome), Table 5 (longitudinal sub-model for PSA outcome) and Table 6 (relative risk sub-model). The parameter estimates for the variance-covariance matrix $\boldsymbol{W}$ from the longitudinal sub-model are shown in Table 3. As described in Appendix A the baseline hazard of the joint model model utilized a cubic P-spline. The knots of this P-spline were placed at the following time points: 0.000, 0.000, 0.000, 0.000, 0.401, 0.801, 1.202, 1.603, 2.003, 2.404, 2.805, 3.205, 3.606, 4.007, 4.407, 4.808, 5.209, 12.542, 12.542, 12.542, 12.542 The parameters of the fitted spline function are given in Table 2.

We present plots of observed DRE versus fitted probabilities of obtaining a DRE measurement larger than T1c, for nine randomly selected patients in Figure 2. Similarly observed versus fitted PSA profiles for nine randomly selected patients are shown in Figure 3.

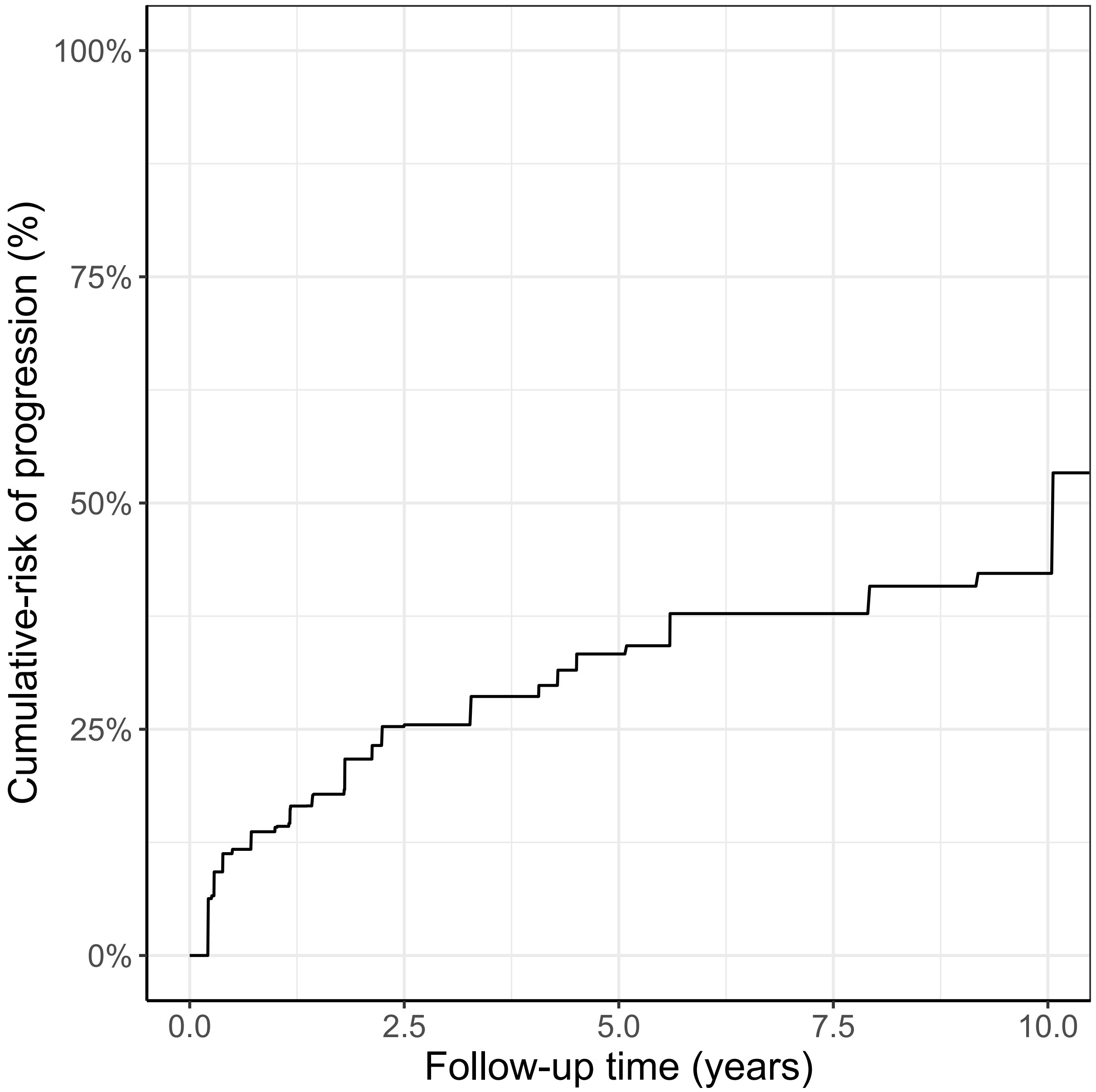

Figure 1: **Estimated cumulative-risk of cancer progression** for patients in the Prostate Cancer Research International Active Surveillance (PRIAS) dataset. Nearly 50% patients (*slow progressing*) do not progress in the ten year follow-up period. Cumulative risk is estimated using nonparametric maximum likelihood estimation [13], to account for interval censored progression times observed in the PRIAS dataset. Censoring includes death, removal from surveillance on the basis of observed longitudinal data, and patient dropout.

Table 2: Estimated parameters of the P-spline function utilized to model the baseline hazard $h_0(t)$ in joint model fitted to the PRIAS dataset. Parameters are named with the prefix 'ps' indicating P-spline parameter.

Variable	Mean	Std. Dev	2.5%	97.5%
ps1	-1.091	0.535	-2.286	-0.235
ps2	-2.113	0.271	-2.638	-1.591
ps3	-2.486	0.308	-3.095	-1.883
ps4	-2.083	0.311	-2.740	-1.483
ps5	-1.918	0.279	-2.460	-1.388
ps6	-2.620	0.265	-3.138	-2.140
ps7	-3.169	0.303	-3.796	-2.580
ps8	-3.416	0.340	-4.075	-2.823
ps9	-3.432	0.345	-4.103	-2.796
ps10	-3.223	0.352	-3.997	-2.573
ps11	-2.840	0.349	-3.577	-2.214
ps12	-2.481	0.350	-3.148	-1.762
ps13	-2.540	0.352	-3.206	-1.840
ps14	-2.841	0.321	-3.447	-2.212
ps15	-3.046	0.381	-3.853	-2.328
ps16	-3.113	0.701	-4.533	-1.796
ps17	-3.195	1.232	-5.894	-0.978

Table 3: Estimated variance-covariance matrix $\boldsymbol{W}$ of the random effects $\boldsymbol{b} = (b_{0d}, b_{1d}, b_{0p}, b_{1p}, b_{2p}, b_{3p})$ from the joint model fitted to the PRIAS dataset.

Random Effects	b_{0d}	b_{1d}	b_{0p}	b_{1p}	b_{2p}	b_{3p}
b_{0d}	9.233	-0.183	-0.213	0.082	0.058	0.023
b_{1d}	-0.183	1.259	0.091	0.079	0.145	0.109
b_{0p}	-0.213	0.091	0.247	0.007	0.067	0.018
b_{1p}	0.082	0.079	0.007	0.248	0.264	0.189
b_{2p}	0.058	0.145	0.067	0.264	0.511	0.327
b_{3p}	0.023	0.109	0.018	0.189	0.327	0.380

Table 4: Estimated mean and 95% credible interval for the parameters of the longitudinal sub-model (4) for the DRE outcome.

Variable	Mean	Std. Dev	2.5%	97.5%
(Intercept)	-4.407	0.151	-4.716	-4.113
(Age – 65)	0.057	0.009	0.039	0.075
$(\text{Age} - 65)^2$	-0.002	0.001	-0.004	0.000
year of visit	-1.089	0.113	-1.292	-0.866

Table 5: Estimated mean and 95% credible interval for the parameters of the longitudinal sub-model (5) for the PSA outcome.

Variable	Mean	Std. Dev	2.5%	97.5%
(Intercept)	2.687	0.007	2.674	2.701
(Age − 65)	0.008	0.001	0.006	0.010
$(\text{Age} - 65)^2$	-0.001	0.000	-0.001	0.000
Spline: [0.00, 0.75] years	0.199	0.009	0.181	0.217
Spline: [0.75, 2.12] years	0.293	0.012	0.269	0.316
Spline: [2.12, 6.4] years	0.379	0.014	0.352	0.406
σ	0.144	0.001	0.142	0.145

Table 6: Estimated mean and 95% credible interval for the parameters of the relative risk sub-model (6) of the joint model fitted to the PRIAS dataset.

Variable	Mean	Std. Dev	2.5%	97.5%
(Age − 65)	0.034	0.005	0.025	0.043
$(\text{Age} - 65)^2$	0.000	0.001	-0.001	0.001
$\text{logit}\big\{\Pr(\text{DRE} > \text{T1c})\big\}$	0.047	0.014	0.018	0.073
Fitted $\log_2(\text{PSA} + 1)$ value	0.024	0.076	-0.125	0.170
Fitted $\log_2(\text{PSA} + 1)$ velocity	2.656	0.291	2.090	3.236

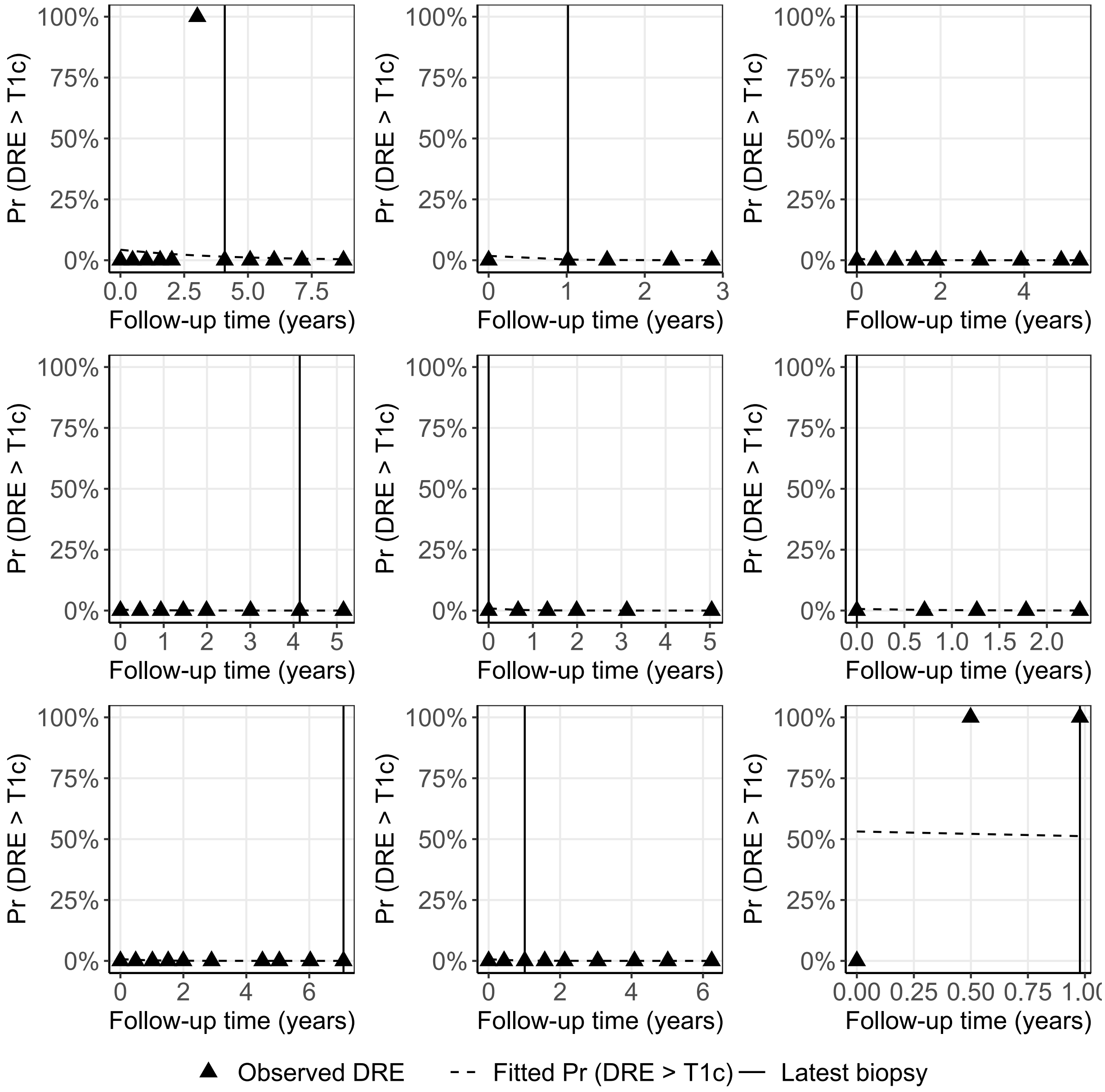

Figure 2: Observed DRE versus fitted probabilities of obtaining a DRE measurement larger than T1c, for nine randomly selected PRIAS patients. The fitted profiles utilize information from the observed DRE measurements, PSA measurements, and time of the latest biopsy. Observed DRE measurements plotted against 0% probability are equal to T1c. Observed DRE measurements plotted against 100% probability are larger than T1c.

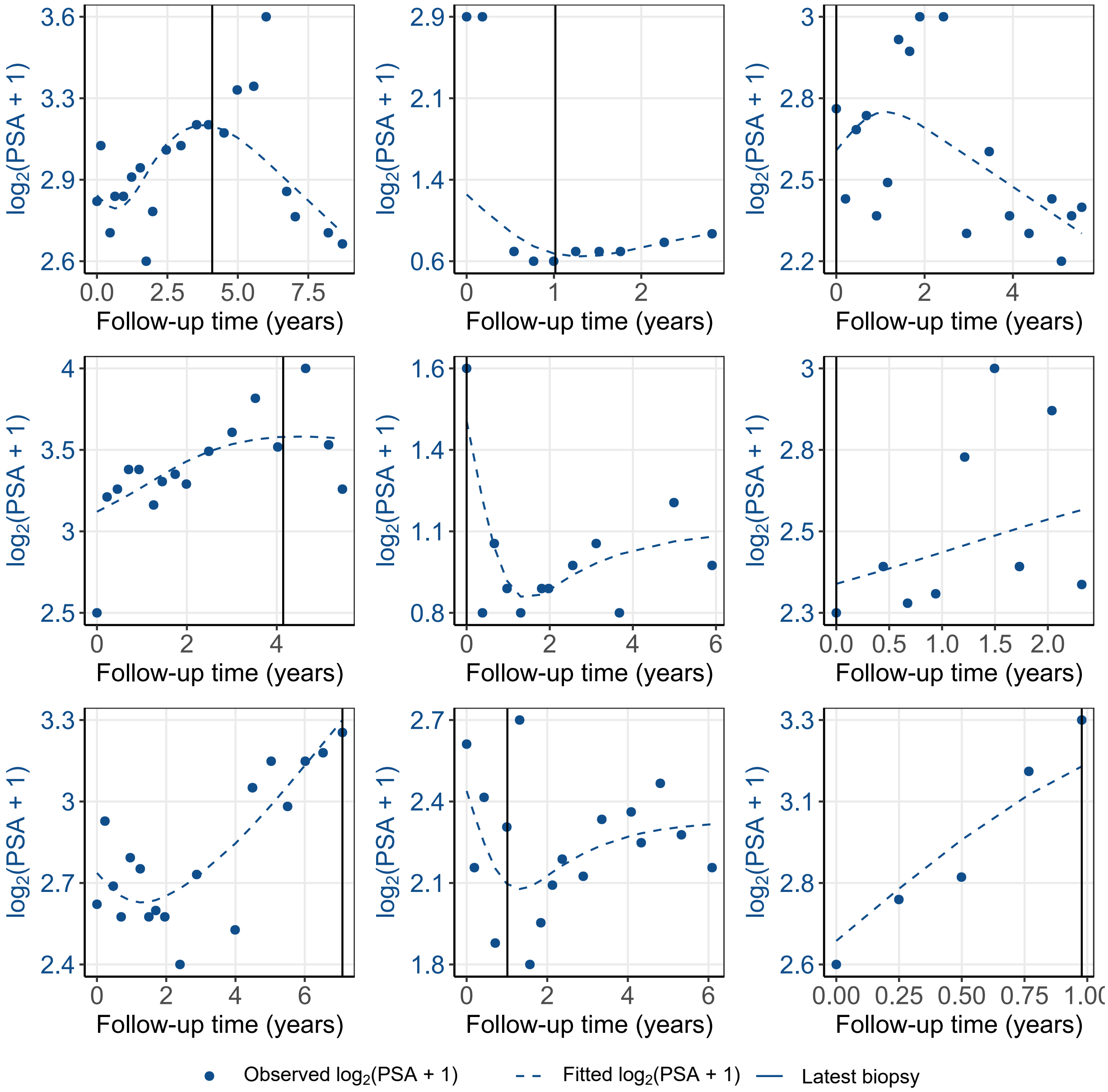

Figure 3: Fitted versus observed $\log_2(\text{PSA} + 1)$ profiles for nine randomly selected PRIAS patients. The fitted profiles utilize information from the observed PSA measurements, DRE measurements, and time of the latest biopsy.

Table 7: Data of the demonstration patient in Figure 5 of the main manuscript. Age of the patient at baseline was 60 years and time of last negative biopsy was 3.5 years. DRE: digital rectal examination.

Visit time (years)	PSA	$\log_2(\text{PSA}+1)$	DRE > T1c
0.00	5.7	2.77	1
0.30	3.2	2.09	-
0.68	4.0	2.30	0
0.97	4.6	2.50	-
1.15	2.9	1.92	0
1.47	3.0	1.95	0
1.77	3.3	2.14	-
2.23	3.5	2.12	0
2.58	4.4	2.39	-
3.21	6.1	2.84	0
3.86	5.9	2.81	-
4.32	3.9	2.31	0
5.00	4.4	2.41	-

B.4 Assumption of t-distributed (df=3) Error Terms

With regards to the choice of the distribution for the error term ε_p for the PSA measurements (5), we attempted fitting multiple joint models differing in error distribution, namely t-distribution with three, and four degrees of freedom, and a normal distribution for the error term. However, the model assumption for the error term were best met by the model with t-distribution having three degrees of freedom. The quantile-quantile plot of subject-specific residuals for the corresponding model in Panel A of Figure 4, shows that the assumption of t-distributed (df=3) errors is reasonably met by the fitted model.

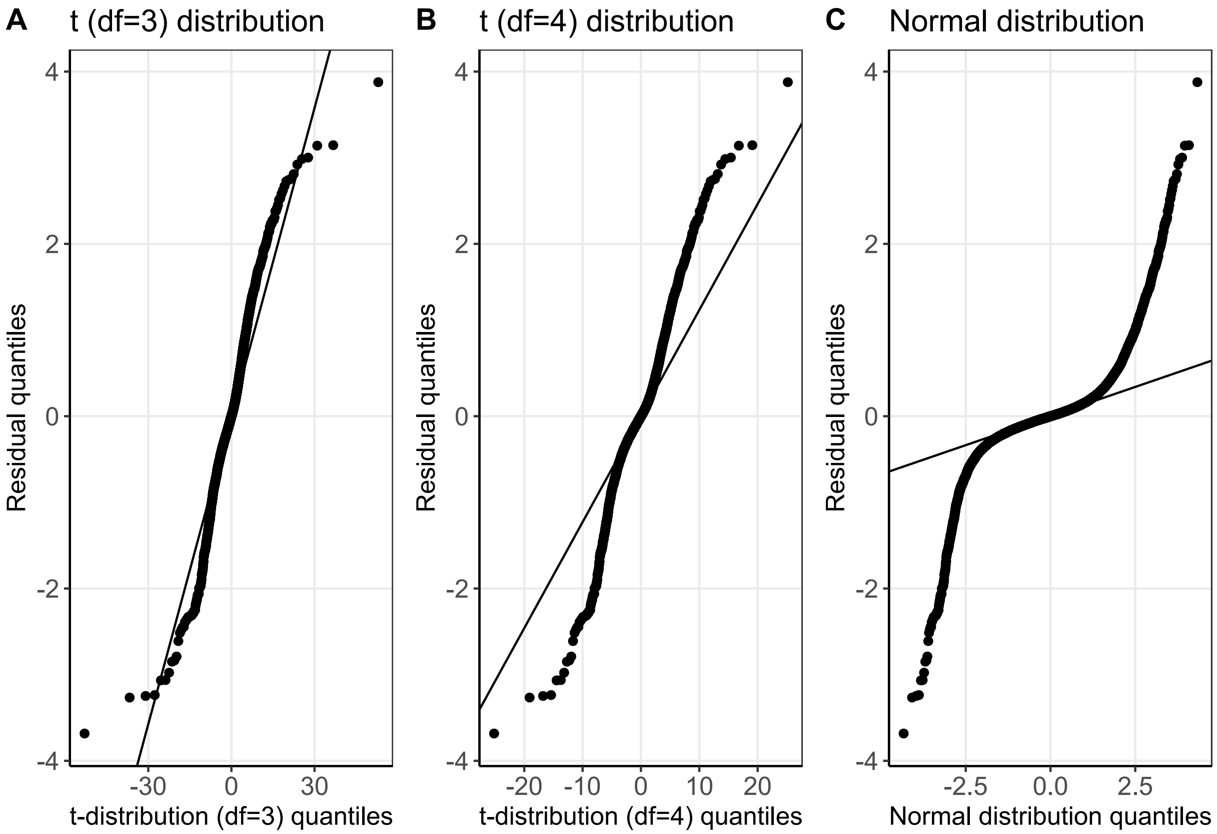

Figure 4: Quantile-quantile plot of subject-specific residuals from the joint models fitted to the PRIAS dataset. **Panel A**: model assuming a t-distribution (df=3) for the error term ε_p. **Panel B**: model assuming a t-distribution (df=4) for the error term ε_p. **Panel C**: model assuming a normal distribution for the error term ε_p.

B.5 Predictive Performance of the PRIAS based Model

We calculate the predictive performance of the PRIAS based joint model using time-dependent area under the receiver operating characteristic curve or AUC (measure of discrimination), and the mean absolute prediction error or MAPE. Mathematical calculations for these in the joint modeling framework are detailed elsewhere [14]. Because these are temporal extensions of their standard versions [15] in a longitudinal setting, at every six months of follow-up (standard visit times in PRIAS), we calculated a unique AUC and MAPE for predicting risk of progression in the subsequent one year (recommended time gap between subsequent biopsies). For emulating a realistic situation, we calculated the AUC and MAPE at each follow-up using only the validation data available until that follow-up. For example, calculations for AUC and MAPE for the time interval year two to year three do not utilize data of patients who progressed before year two. The AUC and MAPE for our model are shown in Table 8.

Table 8: Follow-up time dependent, area under the receiver operating characteristic curves (AUC), and mean absolute prediction error (MAPE), with bootstrapped 95% confidence interval in brackets. The choice of year six as the maximum follow-up period is based on the reasoning that it is roughly the 95-percentile of observed follow-up times.

Follow-up period (years)	AUC (95% CI)	MAPE (95%CI)
0.0 to 1.0	0.658 [0.620, 0.693]	0.234 [0.229, 0.240]
0.5 to 1.5	0.648 [0.631, 0.663]	0.220 [0.213, 0.226]
1.0 to 2.0	0.624 [0.600, 0.644]	0.151 [0.147, 0.155]
1.5 to 2.5	0.649 [0.604, 0.704]	0.127 [0.118, 0.134]
2.0 to 3.0	0.683 [0.629, 0.729]	0.134 [0.121, 0.143]
2.5 to 3.5	0.681 [0.604, 0.739]	0.115 [0.105, 0.128]
3.0 to 4.0	0.647 [0.600, 0.710]	0.079 [0.073, 0.087]
3.5 to 4.5	0.630 [0.583, 0.668]	0.095 [0.089, 0.101]
4.0 to 5.0	0.614 [0.557, 0.659]	0.104 [0.098, 0.111]
4.5 to 5.5	0.615 [0.541, 0.702]	0.101 [0.088, 0.114]
5.0 to 6.0	0.617 [0.550, 0.713]	0.102 [0.086, 0.121]

C Simulation Study

In the simulation study, we evaluated the following biopsy schedules: biopsy every year (annual), biopsy according to the PRIAS schedule (PRIAS), personalized biopsy schedules based on two fixed risk thresholds, namely, $\kappa = 10\%$, and automatically chosen $\kappa^*(v)$ (Section 3 of main manuscript), and automatically chosen $\kappa^*\{v \mid E(D) \leq 0.75\}$ with a constraint of 0.75 years (9 months) on expected delay in detecting progression. The choice of 0.75 years delay constraint is arbitrary and is only used to illustrate that applying the constraint limits the average delay at 0.75 years. We compare all the aforementioned schedules on two criteria, namely the number of biopsies they schedule and the corresponding time delay in detection of cancer progression, in years (time of positive biopsy - true time of cancer progression). The corresponding results, using 500×250 test patients are presented in Table 9. Since the simulated cohorts are based on PRIAS, roughly only 50% of the patients progress in the ten year study period. While, we are able to calculate total number of biopsies scheduled in all 500×250 test patients, but the time delay in detection of progression is available only for those patients who progress in ten years (*progressing*). Hence, we show the simulation results separately for *progressing* and *non-progressing* patients.

Table 9: **Simulation study results for all patients**: Estimated mean (μ), median (Med), first quartile Q_1, and third quartile Q_3 for number of biopsies (nb) and for the time delay (d) in detection of cancer progression in years, for various biopsy schedules. The delay is equal to the difference between the time of the positive biopsy and the simulated true time of progression. Types of schedules: $\kappa = 10\%$ and $\kappa^*(v)$ schedule a biopsy if the cumulative-risk of cancer progression at a visit is more than 10%, and an automatically chosen threshold, respectively. Schedule $\kappa^*\{v \mid E(D) \leq 0.75\}$ is similar to $\kappa^*(v)$ except that the euclidean distance is minimized under the constraint that expected delay in detecting progression is at most 9 months (0.75 years). Annual corresponds to a schedule of yearly biopsies, and PRIAS corresponds to biopsies as per PRIAS protocol.

**Progressing patients (50%)**								
Schedule	Q_1^{nb}	μ^{nb}	Med^{nb}	Q_3^{nb}	Q_1^{d}	μ^{d}	Med^{d}	Q_3^{d}
Annual	1	3.71	3	6	0.29	0.55	0.57	0.82
PRIAS	1	2.88	2	4	0.38	0.92	0.74	1.00
$\kappa = 10\%$	1	2.55	2	4	0.45	1.00	0.85	1.33
$\kappa^*(v)$	1	2.46	2	3	0.45	0.89	0.86	1.26
$\kappa^*\{v \mid E(D) \leq 0.75\}$	1	3.39	3	5	0.32	0.61	0.63	0.88
**Non-progressing patients (50%)**								
Annual	10	10.00	10	10	-	-	-	-
PRIAS	4	6.40	6	8	-	-	-	-
$\kappa = 10\%$	4	4.91	5	6	-	-	-	-
$\kappa^*(v)$	6	6.22	6	7	-	-	-	-
$\kappa^*\{v \mid E(D) \leq 0.75\}$	8	8.68	9	9	-	-	-	-

D Source Code

Source code URL: `https://github.com/anirudhtomer/PersonalizedSchedules`. It has also been uploaded as a zip file with the manuscript.

References

[1] Charles E McCulloch and John M Neuhaus. Generalized linear mixed models. *Encyclopedia of Biostatistics*, 4, 2005.

[2] Elizabeth R. Brown. Assessing the association between trends in a biomarker and risk of event with an application in pediatric HIV/AIDS. *The Annals of Applied Statistics*, 3(3):1163–1182, 2009.

[3] Dimitris Rizopoulos. *Joint Models for Longitudinal and Time-to-Event Data: With Applications in R.* CRC Press, 2012.

[4] Jeremy M.G. Taylor, Yongseok Park, Donna P. Ankerst, Cecile Proust-Lima, Scott Williams, Larry Kestin, Kyoungwha Bae, Tom Pickles, and Howard Sandler. Real-time individual predictions of prostate cancer recurrence using joint models. *Biometrics*, 69(1):206–213, 2013.

[5] Paul HC Eilers and Brian D Marx. Flexible smoothing with B-splines and penalties. *Statistical Science*, 11(2):89–121, 1996.

[6] Eleni-Rosalina Andrinopoulou and Dimitris Rizopoulos. Bayesian shrinkage approach for a joint model of longitudinal and survival outcomes assuming different association structures. *Statistics in Medicine*, 35(26):4813–4823, 2016.

[7] Stefan Lang and Andreas Brezger. Bayesian p-splines. *Journal of Computational and Graphical Statistics*, 13(1):183–212, 2004.

[8] Astrid Jullion and Philippe Lambert. Robust specification of the roughness penalty

prior distribution in spatially adaptive bayesian p-splines models. *Computational Statistics & Data Analysis*, 51(5):2542–2558, 2007.

[9] FH Schröder, P Hermanek, L Denis, WR Fair, MK Gospodarowicz, and M Pavone-Macaluso. The TNM classification of prostate cancer. *The Prostate*, 21(S4):129–138, 1992.

[10] Jay D Pearson, Christopher H Morrell, Patricia K Landis, H Ballentine Carter, and Larry J Brant. Mixed-effects regression models for studying the natural history of prostate disease. *Statistics in Medicine*, 13(5-7):587–601, 1994.

[11] Haiqun Lin, Charles E McCulloch, Bruce W Turnbull, Elizabeth H Slate, and Larry C Clark. A latent class mixed model for analysing biomarker trajectories with irregularly scheduled observations. *Statistics in Medicine*, 19(10):1303–1318, 2000.

[12] Carl De Boor. *A practical guide to splines*, volume 27. Springer-Verlag New York, 1978.

[13] Bruce W Turnbull. The empirical distribution function with arbitrarily grouped, censored and truncated data. *Journal of the Royal Statistical Society. Series B (Methodological)*, 38(3):290–295, 1976.

[14] Dimitris Rizopoulos, Geert Molenberghs, and Emmanuel MEH Lesaffre. Dynamic predictions with time-dependent covariates in survival analysis using joint modeling and landmarking. *Biometrical Journal*, 59(6):1261–1276, 2017.

[15] Ewout W Steyerberg, Andrew J Vickers, Nancy R Cook, Thomas Gerds, Mithat Gonen, Nancy Obuchowski, Michael J Pencina, and Michael W Kattan. Assessing the

performance of prediction models: a framework for some traditional and novel measures. *Epidemiology (Cambridge, Mass.)*, 21(1):128, 2010.
